# Systematic Structural Analyses of Attachment Organelle in *Mycoplasma pneumoniae*

**DOI:** 10.1371/journal.ppat.1005299

**Published:** 2015-12-03

**Authors:** Daisuke Nakane, Tsuyoshi Kenri, Lisa Matsuo, Makoto Miyata

**Affiliations:** 1 Department of Biology, Graduate School of Science, Osaka City University, Sumiyoshi-ku, Osaka, Japan; 2 Department of Physics, Faculty of Science, Gakushuin University, Tokyo, Japan; 3 Department of Bacteriology II, National Institute of Infectious Diseases, Musashimurayama, Tokyo, Japan; 4 The OCU Advanced Research Institute for Natural Science and Technology (OCARINA), Osaka City University, Sumiyoshi, Osaka, Japan; Miami University, UNITED STATES

## Abstract

*Mycoplasma pneumoniae*, a human pathogenic bacterium, glides on host cell surfaces by a unique and unknown mechanism. It forms an attachment organelle at a cell pole as a membrane protrusion composed of surface and internal structures, with a highly organized architecture. In the present study, we succeeded in isolating the internal structure of the organelle by sucrose-gradient centrifugation. The negative-staining electron microscopy clarified the details and dimensions of the internal structure, which is composed of terminal button, paired plates, and bowl complex from the end of cell front. Peptide mass fingerprinting of the structure suggested 25 novel components for the organelle, and 3 of them were suggested for their involvement in the structure through their subcellular localization determined by enhanced yellow fluorescent protein (EYFP) tagging. Thirteen component proteins including the previously reported ones were mapped on the organelle systematically for the first time, in nanometer order by EYFP tagging and immunoelectron microscopy. Two, three, and six specific proteins localized specifically to the terminal button, the paired plates, and the bowl, respectively and interestingly, HMW2 molecules were aligned parallel to form the plate. The integration of these results gave the whole image of the organelle and allowed us to discuss possible gliding mechanisms.

## Author Summary

Human mycoplasma pneumonia, an epidemic of which occurred around the world a few years ago, is caused by a pathogenic bacterium, *Mycoplasma pneumoniae*. This tiny bacterium, about 2 μm long, infects humans by gliding on the surface of the trachea through binding to sialylated oligosaccharides, which are also the binding targets of influenza viruses. The mechanism underlying *Mycoplasma* "gliding motility" is not related to any other well-studied motility systems, such as bacterial flagella and eukaryotic motor proteins. Here, we isolated the internal structure of “attachment organelle", a cellular architecture, and suggested novel component proteins. The organelle was analyzed systematically by focusing on the protein components under fluorescence and electron microscopy, and a possible gliding mechanism was suggested.

## Introduction

Mycoplasmas are parasitic and occasionally commensal bacteria that lack a peptidoglycan layer and have small genomes [1]. *Mycoplasma pneumoniae* is a causative pathogen of human bronchitis and walking pneumonia. Outbreaks of mycoplasma pneumonia occur frequently in many parts of the world, and the increase of macrolide-resistant *M*. *pneumoniae* is a growing problem [2–4]. *M*. *pneumoniae* exhibits gliding motility in the direction of the protrusion at a maximum speed of 1 μm, one-half its cell length, per second [5–8]. This motility, combined with the ability to adhere to epithelial cells, is involved in the pathogenic process, enabling the cells to translocate from the tips of bronchial cilia to the host cell surface [9]. Previous studies, including genome analyses, have shown that this motility is not related to other known mechanisms of bacterial motility, nor does it involve motor proteins known to be involved in eukaryotic cell motility [5, 10–14].

The gliding machinery is a membrane protrusion formed at one pole and can be called an attachment organelle, which is composed of a naplike surface structure and an internal core [15, 16]. The naplike structure corresponds to the complex of P1 adhesin, a plausible leg and also a receptor of sialylated oligosaccharides, which are the ligands on host tissue surfaces [17–19]. The other structure, the internal core, has been known for more than 40 years as a structure that shows rather high contrast in images [20, 21]. Recent studies suggested that this structure can be divided into three parts, including a terminal button, thick and thin paired plates, and a bowl (wheel) complex from the front end [11, 15, 16, 22], and that it is essential for the formation of an attachment organelle but does not include any conventional cytoskeletal proteins such as MreB or FtsZ, unlike other bacterial structures [11, 13]. This structure should be rather stable because it can be isolated on an electron microscopy (EM) grid when the cells are extracted by Triton X-100 [20–23]. However, both detailed and whole images of the internal core are still unclear. Although protein localizations have been shown for four surface proteins, P1 adhesin, P40, P90, and P30, as well as for eight internal proteins, P65, HMW2, P41, HMW1, HMW3, P200, TopJ, and P24, they have not been mapped systematically on the cell images [5, 24]. In the present study, we succeeded in isolating the internal core, observed the details, identified novel protein components, mapped the component proteins of the organelle by protein tagging, and then suggested images for the architecture and the gliding mechanism.

## Results

### Isolation of internal core

**Fig 1:**
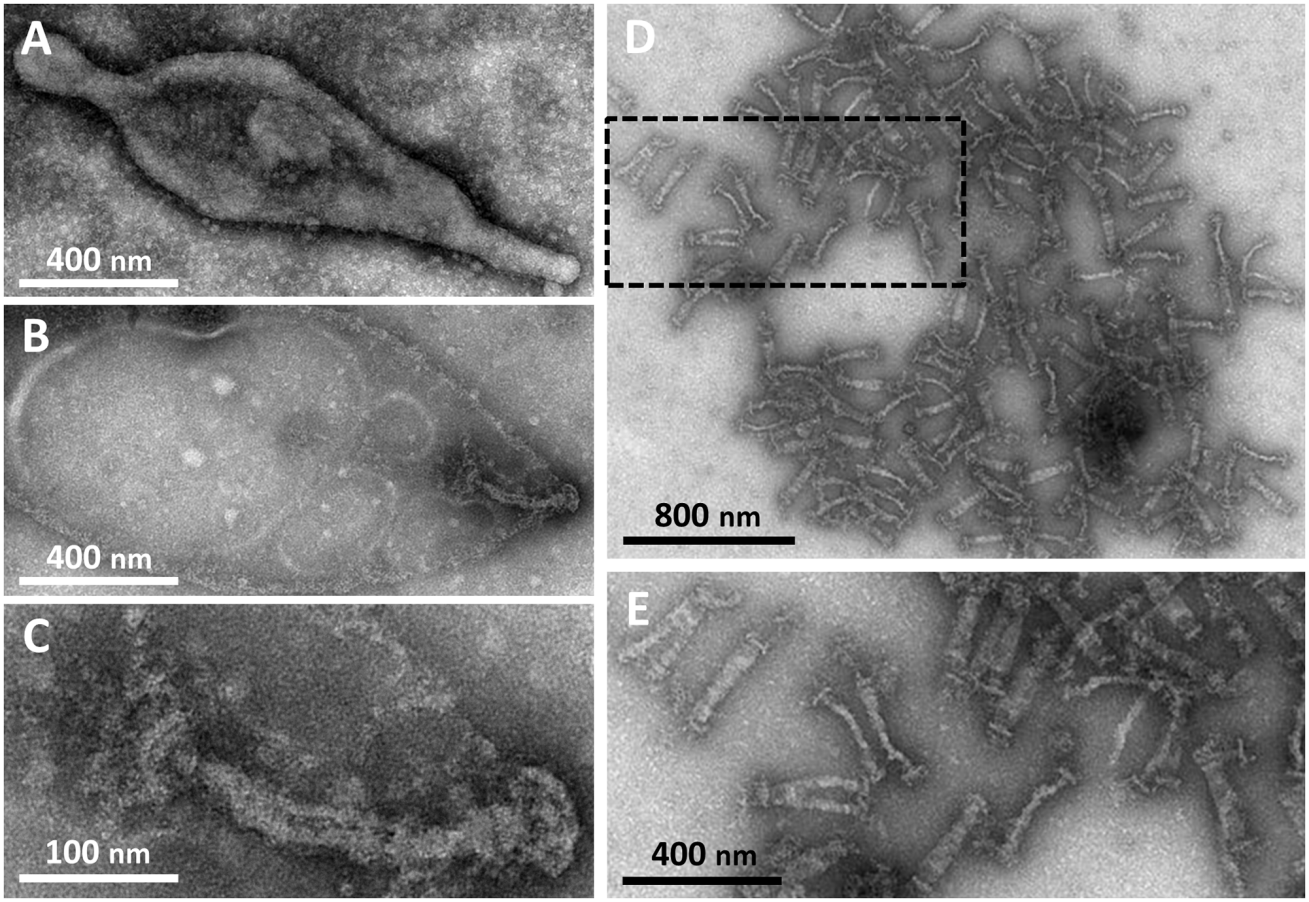
Internal cores of *M*. *pneumoniae* observed by negative-staining EM. (A) Untreated cell bound to a carbon-coated grid. The membrane protrusion at the right pole is the attachment organelle. (B) Cell treated with 1% Tween 20. The cell was treated on a grid. The cell membrane was partially damaged, and the internal core remained at the right pole of the cell. (C) Magnified image of (B). (D) Core fraction isolated through sucrose-gradient centrifugation after cells were treated by 1% extraction of Triton X-100. (E) The boxed area of (D) is magnified. The same magnification was applied to (A), (B), and (E).

To visualize the core in detail and to elucidate the component proteins, we isolated a fraction rich in internal cores. *M*. *pneumoniae* cells recovered from a tissue culture flask were suspended in a buffer, treated with different concentrations of Triton X-100 or Tween 20 for 3 min, and decided the suitable conditions for isolation (Fig 1A, 1B and 1C). The cell fraction treated by 1% Triton X-100 was subjected to stepwise gradient centrifugation, consisting of 0%, 20%, 30%, 40%, 50%, and 60% sucrose layers. After centrifugation, we found a dense layer at the bottoms of the 40% sucrose layers. We recovered and observed the fraction under EM and found that the fraction visually contained only the internal cores, whose features were common to those isolated on a grid, with similar appearances and dimensions, but which did not show other structures such as membrane pieces under EM (Fig 1D and 1E).

### Structural variation of isolated internal cores

**Fig 2:**
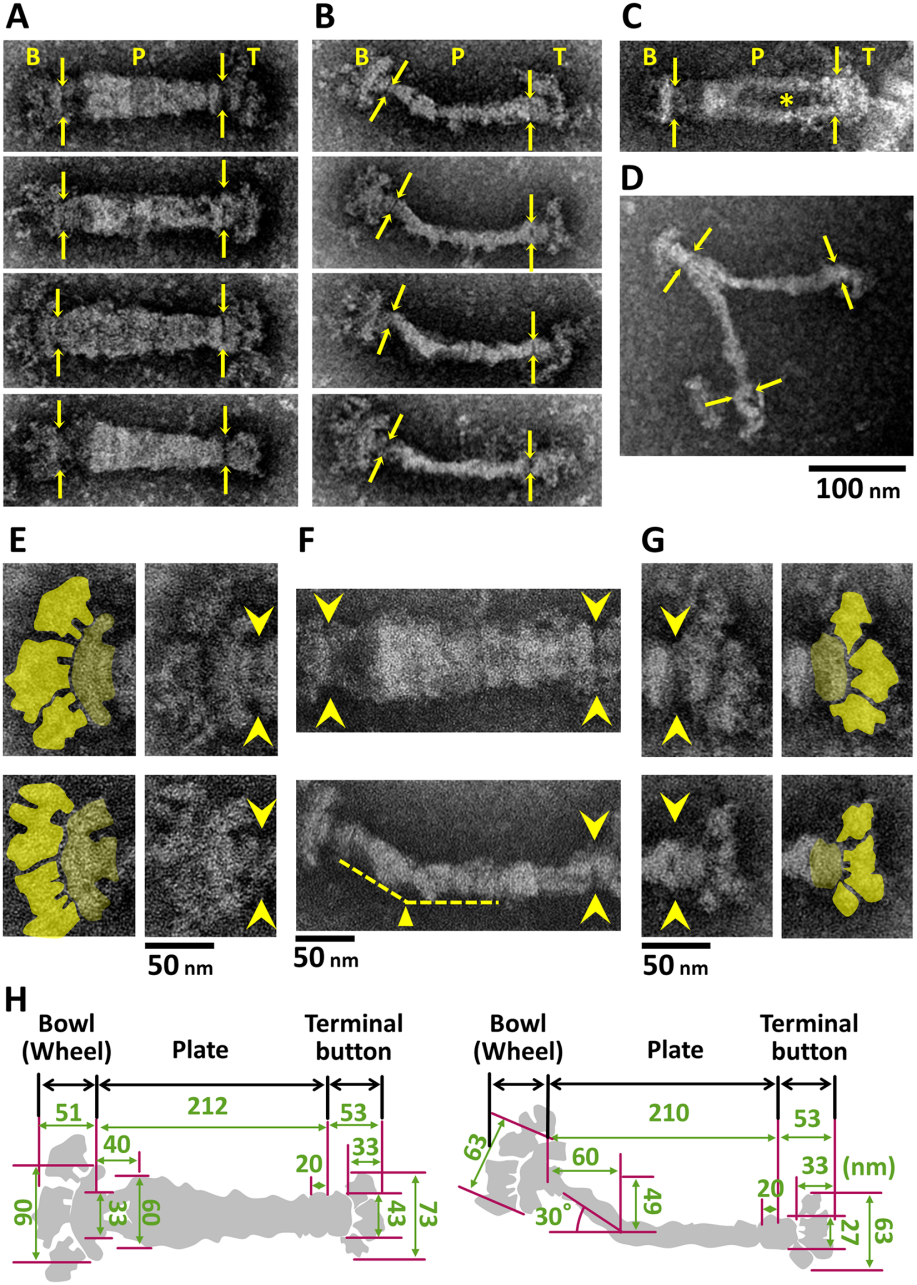
EM images of an internal core. The core can be divided into three parts: a terminal button, paired plates, and a bowl (wheel) complex. Yellow arrows indicate the boundaries between parts. Bold (A), slim (B), fork (C), and branched (D) types are aligned as the cell front on the right. A less-dense area in (C) is marked by an asterisk. (E-G) Structural features of bowl complex. Two typical images are magnified for the bowl (E), the paired plates (F), and the terminal button (G). Yellow arrowheads indicate the boundaries between parts. The original and colored images are shown in the adjacent panels in (E) and (G). Bold and slim structures are shown in the upper and lower panels, respectively. (E) The bowl complex can be divided into two parts, an arch (dark yellow) and accessories (light yellow). (F) The plate in the slim type has a bend around 60 nm from the back end, as marked by the yellow triangle. (G) The terminal button can be divided into a small oval (dark yellow) and accessories (light yellow). (H) Schematics and dimensions of bold (left) and slim (right) types of internal core images. Each length shown is the average of 40 structures.

The images of internal cores allowed us to categorize the cores into four types (Fig 2 and S1 Fig). The "bold" type featured a wide and straight central part (Fig 2A). The "slim" type featured a narrow and bent central part (Fig 2B). The "fork" type featured a lucent part near the center (Fig 2C). The "branched" type featured a branch near the back end of core (Fig 2D). The ratios of these images in the microscopic field were 51%, 39%, 4%, and 6%, respectively. The average lengths of the bold, slim, and fork types were 316 ± 16 (n = 40), 291 ± 11 (n = 40), and 309 ± 18 (n = 10) nm, respectively. As previously suggested, all of the images could be divided into three parts: a terminal button at the front end, central paired plates, and a bowl (wheel) complex at the back end [11, 15, 16, 22]. Both the bowl complex and the terminal button could be divided into two parts (Fig 2E and 2G). The backside part of bowl complex has not been visualized in previous studies [15, 16, 20, 21]. Although the structures at both ends were common among all four types, the central "plate" was specific to each type. In the bold type, the plate was rather symmetrical around its axis, with a maximum width of 60 ± 10 nm (n = 40). In the slim type, the narrow plate had a bend around the position at 40–60 nm from the back end (Fig 2H). The plate of the fork featured a low-density area near the center. In the branched type, the plate was branched near the bowl complex with duplicated terminal buttons. In the isolated core fractions observed here, the detailed structures were visible more clearly than the structures of the cells treated on EM grids as shown in Fig 1, probably because the structures covering the internal cores were removed in the isolated fraction.

### Protein components of internal cores

**Fig 3:**
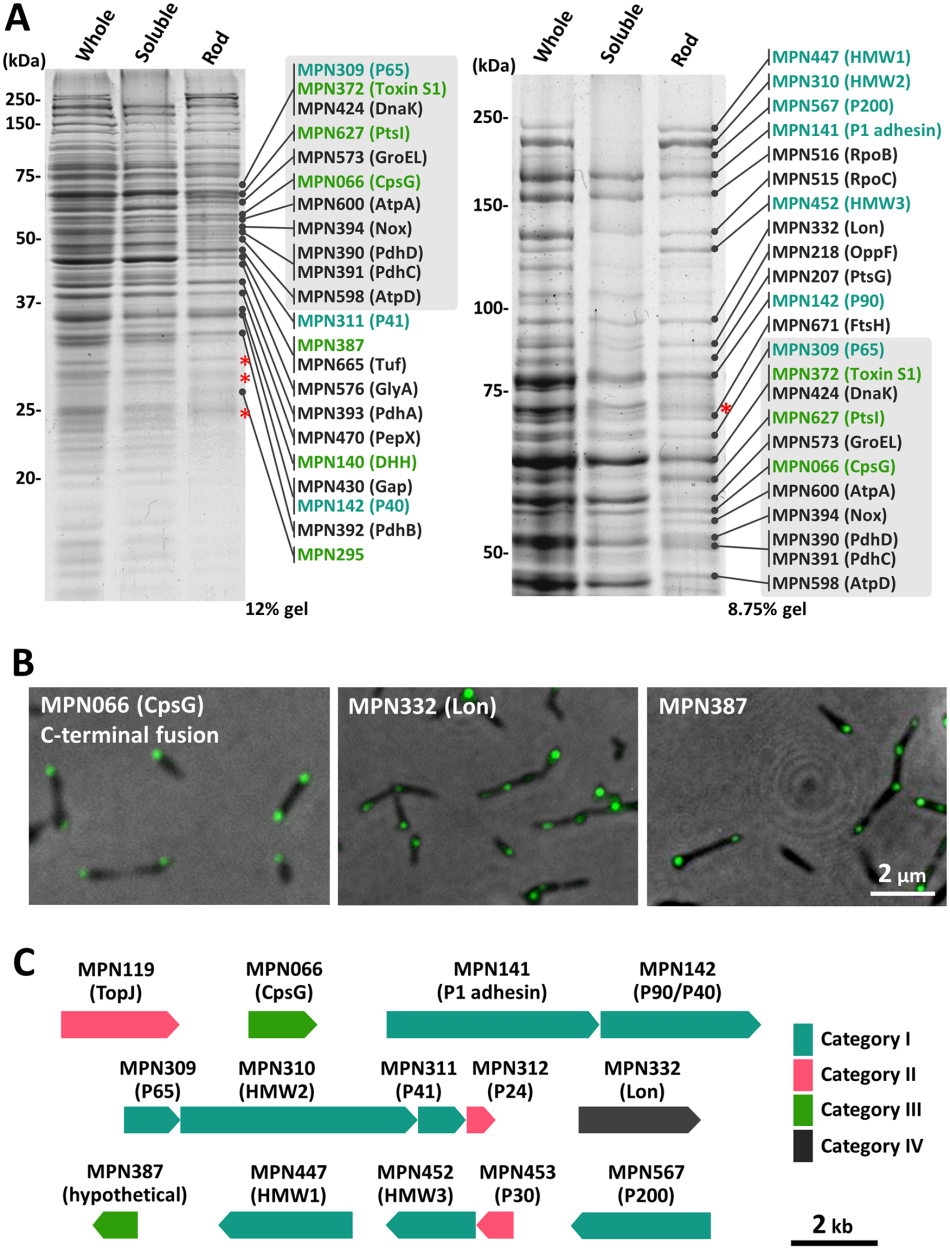
Protein components of an internal core. (A) Protein profiles of the core fraction. The cells were treated by Triton X-100, fractionated by sucrose-gradient centrifugation, subjected to SDS-PAGE, and stained by CBB. Gel images of 12% and 8.75% polyacrylamide are shown in the left and right panels, respectively. The amount of fraction applied to each lane was adjusted to derive from the same number of cells. The molecular mass is shown on the left. The protein bands identified by PMF are marked by an arrow with the gene ID (MPN number) and the annotation [13]. The asterisks indicate the bands that were not identified by PMF. Gray boxes highlight the identical protein bands identified from the gels with both acrylamide concentrations. The gene ID and annotations are colored according to their categories (see the text). (B) Cell images labeled by EYFP for MPN066, MPN332, and MPN387. Phase-contrast and fluorescence images are merged. The cell images labeled for other proteins are shown in S2 Fig. (C) ORFs encoding the components of the attachment organelle.

The proteins involved in the core fraction were identified by peptide mass fingerprinting (PMF) (Fig 3) using the following procedure. (i) The proteins of the whole cell lysate, soluble, and core fractions were developed by SDS-PAGE and applied to Coomassie Brilliant Blue (CBB) staining, in which the band density is better related to the protein amount than other staining methods [25]. (ii) Thirty-four bands isolated from the lane of the core fraction were identified by PMF. (iii) To identify the proteins specific to the core fraction, the protein bands detected at the same position in the lane of the soluble fraction were also identified by PMF. If these peptides were derived from different proteins or if no bands were found at the corresponding position in the soluble fraction, the protein from the band was defined as specific to the core fraction. (iv) To examine the possibility that an apparently single band contains more than one proteins, the Mass Spectrometry signals which were not assigned to the identified proteins were subjected to the next identification.

Finally, we identified 34 proteins from the core fraction and listed 37 proteins as components of the attachment organelle and candidates in four categories, as follows (S1 Table). (Category I) Nine proteins identified in the present study were already known as the components of the attachment organelle: P1 adhesin, P90, P40, P65, P41, HMW1, HMW2, HMW3, and P200 [5, 26]. P1 adhesin, P90, and P40 were found also in the soluble fraction, suggesting that a fraction of these proteins might be solubilized by the detergent because they localized on the cell surface [18]. (Category II) Three proteins, TopJ, P24, and P30, were not found in the core fraction, although they have been reported as the organelle components [5]. These proteins may detach from the attachment organelle easily, or they may exist in smaller amounts than the other component proteins. (Category III) Six proteins were found specifically in the core fraction. (Category IV) Nineteen proteins were found in both the core and soluble fractions. The molar ratios of proteins specific to the core fraction were determined by band densitometry. The previously reported component proteins were found in higher protein amount than the others.

### Subcellular localization of component proteins visualized by fluorescence protein tagging

We listed 37 proteins in S1 Table, composed of 12 proteins previously reported as a component of the attachment organelle (categories I and II) and 25 proteins found in the core fraction in the present study (categories III and IV). Six proteins in category IV, PtsG (MPN207), OppF (MPN218), PdhB (MPN392), PdhA (MPN393), GlyA (MPN576), and Tuf (MPN665), have been known to be a part of large complexes involved in cell metabolism, which likely remained in the core fraction [27]. Therefore, we focused on the other 19 proteins in categories III and IV as candidates for novel components of the attachment organelle. To examine the involvement of these 19 proteins in the attachment organelle, we applied EYFP fusion tagging to 31 proteins including categories I and II (S2A Fig) [7, 28]. In this system, the *M*. *pneumoniae* genes of interest were fused by the *eyfp* gene at their N-termini by using the Gateway subcloning method, as described previously [7]. The fused genes were delivered into random positions on the *M*. *pneumoniae* chromosome using the Tn*4001* transposon vector system [29], and expressed in the wild-type background by a strong promoter of the *tuf* gene [7]. First, we applied this strategy to all 12 proteins in categories I and II, which are known components. As shown in the legend of S2 Fig, the localizing patterns of the EYFP signal were divided into four types: type "a" had a focused signal at the attachment organelle, type "f" had focused signals at different positions than the organelle, type "d" had a diffuse signal in the whole cell, and type "n" had no signal. The EYFP fusions with P65 (MPN309), HMW2 (MPN310), P41 (MPN311), HMW1 (MPN447), HMW3 (MPN452), and P24 (MPN312) showed clear localization at the organelle (type "a"), consistent with previous reports [7, 12, 30–32]. P200 (MPN567) and TopJ (MPN119) showed localization at the organelle (type "a"), but also additional localization elsewhere (type "f"). Probably, this was caused by the excess of proteins produced from the strong promoter, *tuf*, stacked in a space in a cell usually at the other end of the organelle. The expression of any EYFP fusions did not show obvious defects in the binding or gliding activities of *M*. *pneumoniae*. At room temperature (RT), less than 1.3% of cells (n = 150) attached to the glass bottom dish glided with gliding speeds less than 0.01 μm/s, 40 times slower than those at 37°C. We then took pictures at RT without fixation. P1 adhesin (MPN141) and P30 (MPN453) showed diffuse signals (type "d"), although they are known to localize at the attachment organelle as shown by immunofluorescence microscopy [12, 31, 32]. Probably, this was caused by the steric effect of EYFP fusion to the N terminus on protein folding or sorting. We next examined the C-terminus fusion and found that the signals of P1 adhesin and P30 were focused at the attachment organelle, suggesting that the fusion site is critical in some cases [7, 28, 30, 33, 34]. MPN142 is known to be cleaved into two proteins, P40 and P90, which are composed respectively of 1–454 and 455–1218 amino acid residues after translation and which localize at the attachment organelle [12, 31, 32, 35]. We tried EYFP fusion to both sides of MPN142 and to the N terminus of the P90 sequences. The N-terminal tagging of MPN142 showed diffuse subcellular localization, and SDS-PAGE showed that the cleavage of MPN142 did not occur. The C-terminal tagging of MPN142 and the N-terminal tagging of the P90 part of MPN142 showed no and diffuse fluorescence in a cell, respectively.

### Subcellular localization of candidates for organelle components examined by fluorescence protein tagging

Next we examined the subcellular localization of the 19 candidate proteins in categories III and IV by N-terminal fusion and found that 2 proteins, MPN387 and MPN332, localized at the attachment organelle (Fig 3B). MPN387, found specifically in the core fraction, is featured in its sequence by a coiled-coil domain spanning 87–285 amino acid residues in the total of 358. MPN332, found in both the core and soluble fractions, has been annotated as Lon protease, an ATP-dependent protease [13]. C-terminal fusion was also applied to the proteins found specifically in the core fraction, including MPN066, MPN140, MPN295, MPN372, and MPN627, revealing that MPN066, annotated as "CpsG", phosphomannomutase, or phosphoglucomutase [13], localizes at the organelle. On the basis of these results, we concluded that the attachment organelle includes three additional novel components: MPN066, MPN387, and MPN332. MPN332 was detected also in the soluble fraction, suggesting that this protein departs from the attachment organelle easily or exists abundantly. In the PMF, four proteins were identified specifically in the core fraction but were not proven to localize at the attachment organelle by EYFP fusion tagging:MPN140 (ORF4), MPN295, MPN372 (CARDS toxin), and MPN627 (PtsI). Namely, the fluorescence images of MPN140, MPN372, and MPN627 showed diffuse patterns, and the signals of MPN295 focused on positions other than the attachment organelle. These proteins may be involved in other protein complexes that behaved similarly to the internal core in the fractionation by centrifugation. Otherwise their localization to the attachment organelle may be affected by the EYFP fusion.

To address the possibility that additional focal localization was caused by heterogeneity in the expressed fusion proteins, we detected the fusion proteins for P200 (MPN567), TopJ (MPN119), MPN295, MPN390 (PdhD), and MPN391 (PdhC) by Western blotting using an antibody against EYFP (S3 Fig). The major bands were detected around the positions expected from the amino acid sequences. These observations suggest that the focal localization at positions other than the organelle was not caused by the heterogeneity in expressed proteins.

### Mapping 13 component proteins by fluorescence microscopy

**Fig 4:**
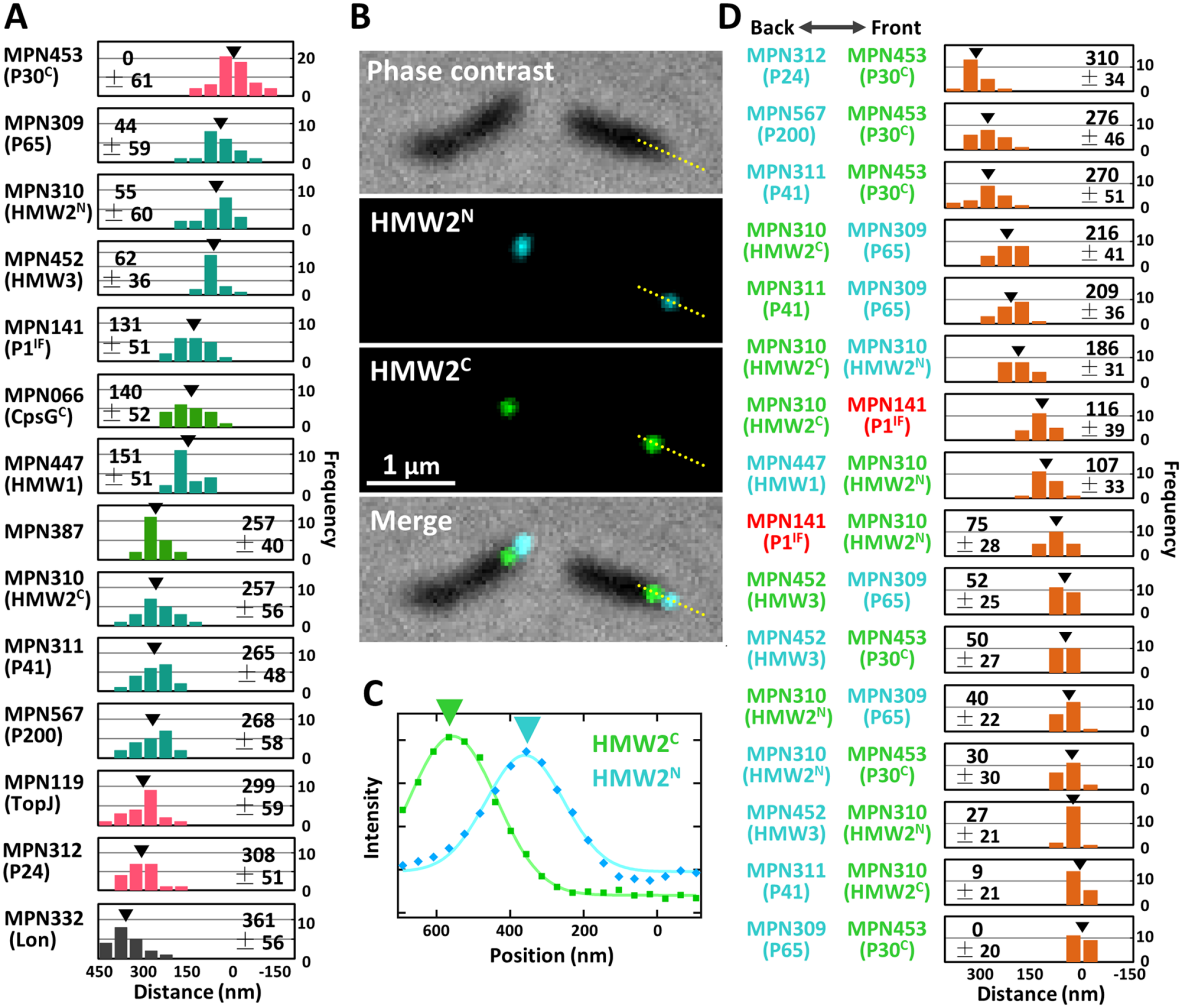
Localization of attachment organelle components determined by fluorescence in a living cell. (A) Distribution of peak positions of fluorescence relative to the cell front. The cell front was defined from phase-contrast images as shown in S5 Fig. Gene identification number (ID) and protein annotation are indicated on the left. The average from 20 cells is marked by a black triangle and shown with standard deviation (SD) in each panel. The histograms are colored according to their categories shown in Fig 3 and S1 Table. (B) Fluorescence of EYFP fused to the C terminus and of ECFP fused to the N terminus of HMW2 in a cell. Fluorescence and phase-contrast images are merged. (C) Measurement of relative positions of EYFP and ECFP signals. Signal intensities along the yellow dotted line in (B) were plotted and traced as Gaussian distribution. (D) Distribution of the distances between two different proteins. Gene ID and its annotation are presented on the left with colors showing their fluorescence; EYFP (green), ECFP (cyan), and Cy3 (red). P1 adhesin was detected by immunofluorescence (IF) microscopy using a monoclonal antibody [32]. The average of 20 cells is shown with SD and marked by a black triangle in each panel.

To obtain a clear image of the attachment organelle, we tried to map the fluorescent foci of the 13 component proteins tagged by EYFP, on an attachment organelle that was about 450 nm long (Fig 4). The fluorescence intensity of a cell with a typical extended shape was profiled along the cell axis and its peak position was determined (S4 Fig). The distribution range depended on the proteins, i.e. P1 adhesin spread more than P30, HMW3, HMW1, and P65, as reported previously [31, 32]. To map the fluorescent foci on the cell axis precisely, the phase-contrast image was used to determine the front end position of a cell, assuming that the attachment organelle is uniform in shape and density among individual cells (S5 Fig). The cell image density was normalized by a control object, a 200-nm polystyrene bead as the 100% density, because the image density depends on the illuminating conditions generally in bright-field optical microscopy. As P30, a transmembrane protein [36] was found to localize at the front side, the position of this protein along the cell axis corresponding to 16 ± 5% cell image density (n = 60) was set as the zero position. Therefore, we mapped the axial peak positions of fluorescence as the distance from the position where the image density of cell was 16% of that at the bead center. This process was applied to 20 cells, each labeled for the 13 proteins, and the average positions were mapped (Fig 4A). Note that these histograms show the variations in peak positions of cells, not the signal distributions in a single cell which is shown in S4 Fig.

The analysis of the amino acid sequence of HMW2 suggests that 11 regions, composed of 1257 amino acid residues of the whole 1818 residues, form a coiled-coil structure [37]. There have been conflicting data regarding the alignment of HMW2 molecule in the internal core, namely parallel [37, 38] or perpendicular [39] to the core axis. If the molecules are aligned parallel, the total length is predicted to be 190 nm. Therefore, we fused EYFP to the C-terminus of this gene and analyzed the signal positions. The signal labeled at the C-terminus was localized at 257 ± 56 nm from the front, and the signal at the N-terminus was localized at 55 ± 60 nm, suggesting that HMW2 molecules are aligned in parallel along the cell axis, pointing the N terminus to the front (Fig 4B and 4C). The 93% and 95% of isolated fluorescent foci were found in the organelle for N-terminus and C-terminus fusions, respectively (S3 Fig). The Western blotting analyses showed the major bands at the positions close to those of 245 and 249 kDa expected from the amino acid sequences, respectively for the N-terminal and C-terminal fusions. The internal translation product corresponding to amino acids 1620–1818 of HMW2, designated as P28 was also detected near the expected gel position with the relative protein amount to HMW2 consistent with the previous study [40, 41]. These results suggest that the fusion proteins were expressed and incorporated into the organelle in manners similar to those of the original HMW2 and P28, because the signals were not detected in additional positions. If this is the case, the part of HMW2 corresponding to the P28 may be responsible for the localization of the C-teminal part. All sets of data pairs were examined by ANOVA test (S3 Table) and the proteins were classified into six clusters as summarized in S6 Fig. Next, the relative positions of 16 pairs of proteins were also examined, by measuring the distances between fluorescent foci labeled by different fluorophores, including EYFP, ECFP, and Cy3 (Fig 4 and S2 Fig). The distances between the positions within all 16 pairs were in good agreement with the values expected from the positions of the individual proteins, showing that the mapping of individual proteins using the density of phase-contrast image is reliable.

### Mapping six component proteins by electron microcopy

**Fig 5:**
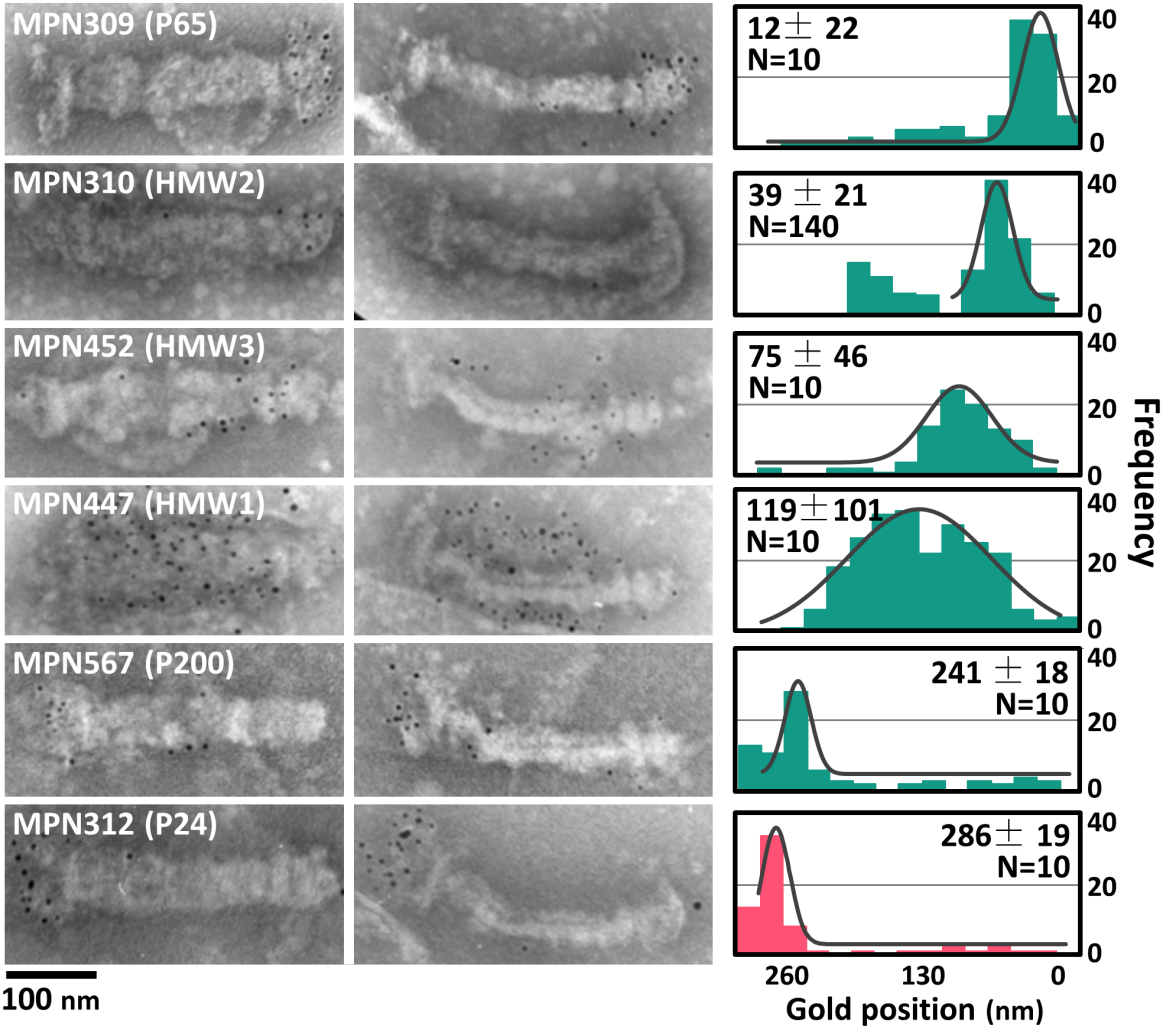
Localization of attachment organelle components determined by immunogold EM. The cells expressing N-terminus EYFP-fusion proteins were treated by 0.3% Triton X-100, chemically fixed, and sequentially labeled by antiserum against EYFP and the secondary antibody conjugated with a 5-nm gold particle. Bold and slim core structures are shown in the left and the right panels, respectively, for each protein. Histograms show the distributions of the gold positions measured along the axis and these positions are traced by Gaussian distribution shown by a black line. The bar of the histogram is colored according to the categories shown in Fig 3. The peak positions from the terminus and the number of examined cores are shown in each panel.

**Fig 6:**
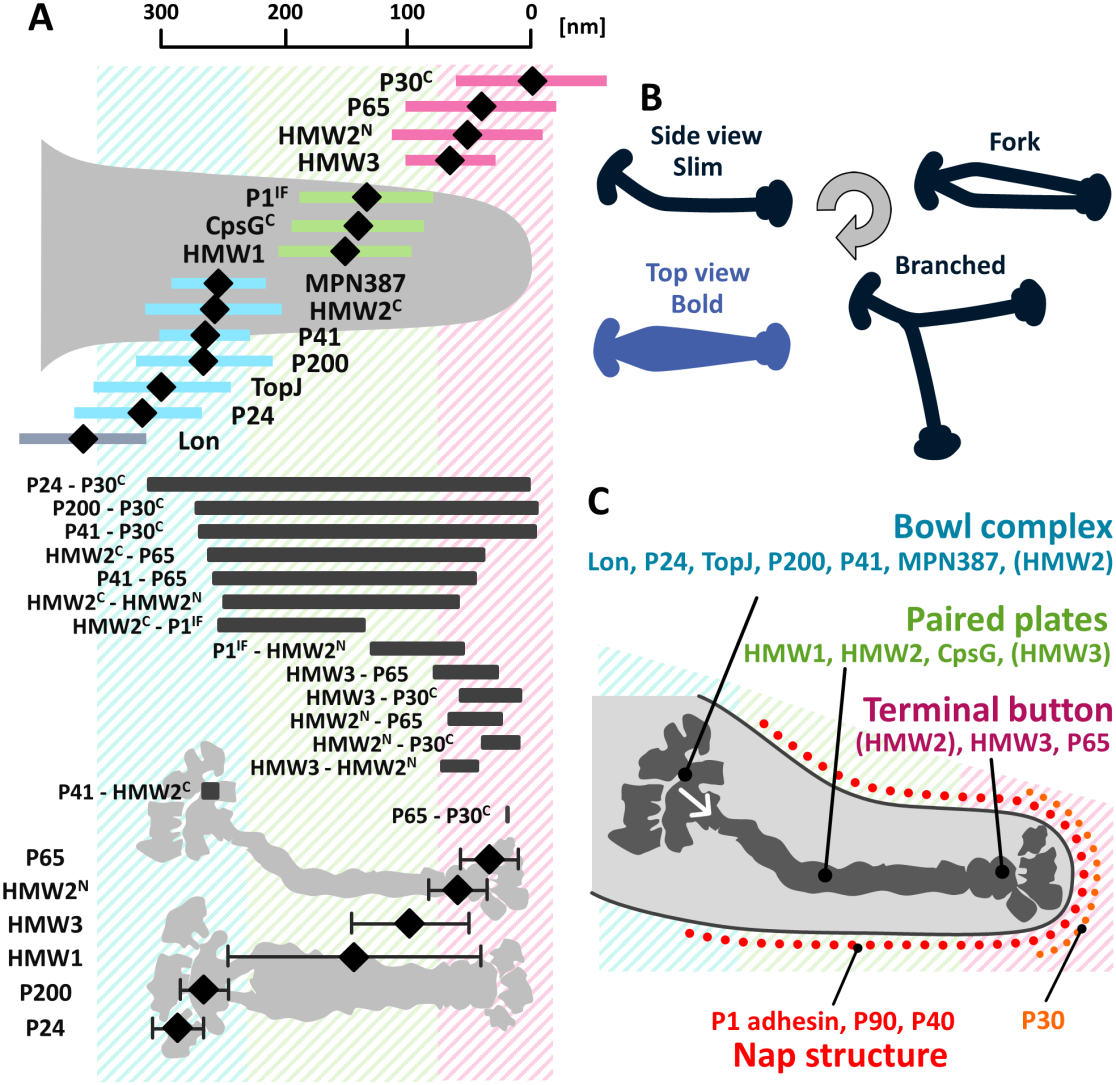
Schematic illustrations of the attachment organelle. (A) Upper: Positions of internal proteins along attachment organelle determined from EYFP fluorescence. The average position in individual cells and SD relative to the cell edge are plotted with a black diamond and a colored bar, respectively. The proteins are mapped onto three localization groups, the terminal button (pink), the paired plates (green), and the bowl complex (blue). Middle: Distance between two signals measured from fluorescence microscopy. The positions of the terminal button, paired plates, and bowl complex are colored magenta, green, and cyan, respectively. Lower: Positions of internal proteins along a core determined from immunogold EM. The calculated peak and SD are presented as a black diamond and a bar, respectively. (B) Relations among four types of core structures. The slim, the fork, and the branched types are aligned to the EM grid in the common way. A possible reproduction cycle of an internal core is suggested by the arrow. (C) The attachment organelle including component proteins. The force is generated at the bowl complexes, is transmitted through the paired plates as indicated by a white arrow, and reaches the P1 adhesin complex.

To map those positions onto EM images, immunogold-EM was applied. The cells labeled by EYFP fusion for 13 protein constructs were attached to an EM grid, treated with Triton X-100 to expose the internal core, and then labeled with antiserum against EYFP conjugated by 5 nm of colloidal gold (Fig 5). P1 adhesin was not examined because it is anchored to the cell membrane which is removed by the detergent treatment [18]. The images of internal cores were less clear than those observed in Figs 1 and 2, and S1 Fig, plausibly due to the labeling process by the antibodies. Specific labeling by gold was detected for six constructs, P65, HMW2 (N-terminus fusion), HMW3, HMW1, P200, and P24, around the internal core, but the other seven constructs, HMW2 (C-terminus fusion), P41, TopJ, P30, CpsG (MPN066), MPN387, and Lon (MPN332) did not show specific gold labeling. The structure and assembly of the proteins successfully detected in this manner may be suitable to be exposed by the Triton X-100 extraction, because the fused EYFP was detected by the antibody. On the other hand, the proteins unsuccessfully detected in this manner may be removed by the detergent or they may not be exposed even after the detergent treatment. The gold positions were plotted along the long axis, fitted by Gaussian distribution, and mapped along the cell axis (Fig 5). Most of those positions were in good agreements with the fluorescence images (Fig 6A). The position of HMW3 did not agree well with that from fluorescence, suggesting that this protein was slightly detached from the core by the Triton treatment for immuno electron microscopy. The distribution of gold labeling showed the following localization: P65, terminal button; HMW2 (N-terminus), front of paired plates; HMW3, front side of paired plates; HMW1, paired plates; P200 and P24, bowl complex.

## Discussion

### Electron microscopy

In the present study, the internal core was fractionated through Triton X-100 treatment and centrifugation, and then observed by EM (Fig 1). This procedure gave us images with higher contrast and lower backgrounds than those in previous studies without fractionation [6, 15, 16, 20, 21]. In a previous study, a similar approach was applied to the internal core of *M*. *pneumoniae* [23]. However, the sucrose-gradient centrifugation applied in the present study allowed us to isolate internal cores with less damages to the structure than occurred in the previous study (Fig 2 and S1 Fig). In the present images, the isolated core structure included the bowl complex, which could be observed only in the electron cryotomography (ECT) of *M*. *pneumoniae*, and negative staining of Triton extracted *M*. *gallisepticum*, a species related to *M*. *pneumoniae* [6, 15, 16]. The images obtained here were clear enough to determine the detailed dimensions for the first time [15, 16, 20, 21], but not uniform enough for image averaging for higher resolution, suggesting that the internal core has some flexible parts. This feature is shared with the internal structure of *M*. *gallisepticum* [6].

How are the four types of images obtained in the present study related to one another (Fig 2 and S1 Fig)? Previous analyses by fluorescence microscopy showed that the attachment organelle replicates for cell reproduction[24, 30, 32]. On the basis of this observation, the possible relationships among these four types can be traced from the slim type, which is probably a side view of an internal core in a cell (Fig 6B). Possibly, the fork type may be a partly duplicated form of the slim type, where the daughter plates are two-storied. The branched type may be the later stage of replication. The attachment organelle has been suggested to replicate prior to cell division, based on the duplication scheme of the fluorescence signals labeled to the component proteins, and the frequency of branched type observed in the present study (4%) is consistent with the previous observations showing that 5.5% of growing cells have two signals of the attachment organelle at adjacent positions [30, 32]. The bold type is the top or bottom view of these three forms. In the internal structure of *M*. *gallisepticum* extracted from a cell by Triton X-100 also, an image similar to the branched type was observed with a frequency comparable to the frequency of cells with two organelles under optical microscopy [6], suggesting that the replicating scheme is conserved in the related species. However, additional experiments are needed to conclude this reproduction scheme, because the fork and branched structure could be converted from the bold type in the core isolation process. Previous ECT imaging of the whole cell showed that the middle part of the internal structure is composed of thick and thin plates [15, 16]. In the present study, however, the plate was not resolved into two layers in the core fraction (Figs 1 and 2), suggesting that the thin plate was removed in Triton treatment or the paired plates stick together in the dried condition of negative staining. Similarly, the distal ends of terminal button and bowl appeared differently from those in ECT [15, 16], namely some accessories were observed in the present study (Fig 2). Although the accessories did not show uniform shapes, they were observed reproducibly. Probably, these structures were exposed and enhanced for contrast by the detergent extraction and the staining by molybdate. We should consider that the negative staining EM after extraction and isolation may tend to give images with higher contrast and more changes from the original than those from ECT.

### Newly identified component proteins

In a previous study, *M*. *pneumoniae* cells were extracted by Triton X-100 and analyzed by mass spectrometry to identify the protein components of the insoluble fraction [23]. Our present study adopted a similar strategy but contains three critical improvements, as follows (Fig 3). (i) The proteins in the soluble fraction were also identified as reference. (ii) The analyzed fraction was well isolated from others and did not contain other structures based upon examination by EM. (iii) Identified proteins were further analyzed by EYFP tagging. In the present study, 25 proteins were suggested as candidates for novel core components by PMF, and then MPN066, MPN332, and MPN387 were suggested to be components by EYFP tagging (Fig 3C). MPN387 is conserved among the genomes of related species, *M*. *genitalium* and *M*. *gallisepticum* [42, 43]. This protein has been suggested to be involved in the gliding mechanism, because the depletion of this protein by a transposon insertion induced a nongliding phenotype, retaining hemadsorption activity [44]. MPN387 localized around the bowl complex (Figs 3B and 4), in common with P200, which is involved in movement rather than binding [45]. These findings suggest that the bowl complex has a critical role in force generation for gliding. The other two proteins, MPN066 and MPN332, have annotations to proteins widely distributed in bacterial genomes [13]. MPN066 is CpsG, a phosphomannomutase or phosphoglucomutase involved in sugar metabolism [46]. MPN332 is a Lon protease which have a role in diverse cellular functions in bacteria [47, 48]. The roles of these proteins in cytadherence and gliding are unclear. It has been suggested that the attachment organelle may have roles in addition to adhesion and gliding, because usually the replication of this organelle occurs as an early event in the cell division process [30, 32]. We found three proteins as novel components of the attachment organelle, on the basis of the EYFP tagging results. However, EYFP fusion sometimes has steric effects on the folding and sorting of the proteins of interest [7, 28]. Therefore, the involvements of other candidates to the attachment organelle cannot be ruled out, especially for the proteins in category III.

### Architecture of attachment organelle and gliding mechanism

Now, 15 proteins have been assigned as components of the attachment organelle, including three newly identified proteins. We then systematically mapped 13 proteins that were available for EYFP tagging, and six of those 13 proteins also on EM images, and suggested an integrated image for the attachment organelle which is consistent with previous studies, as follows (Fig 6A and 6C). (i) The terminal button is composed of P65 and HMW3 [31, 32]. HMW3 localize at the boundary with the paired plate. (ii) The paired plates are composed of HMW1, HMW2, and CpsG [31, 32, 38]. HMW2 molecules are aligned in parallel along the axis with the N terminus at the front extending into the terminal button and the bowl complex [37, 38]. HMW1 localizes at distant positions from the paired plates as observed in the slim core EM images (Fig 5), which is consistent with the "peripheral association" which was reported previously [49]. (iii) The bowl complex is composed of MPN387, P41, P200, TopJ, P24, and Lon protease [7, 45, 50]. MPN387 and P200 are known to be necessary for gliding rather than binding [30, 45]. P41 has a role in connecting the organelle with other parts but is not involved in the gliding itself, because the organelle tends to glide away leaving other parts of the cell [51]. (iv) On the surface, P1 adhesin is distributed around the core with P40 and P90 [32, 52–54]. P30 is localized at the surface of the front end [32, 36]. This mapping is consistent with the protein clustering based on the fluorescence position and ANOVA test, while some of protein pairs both mapped on the terminal button or the bowl complex were supported for the significant difference in their distribution even in the same localization group (S3 Table, S6 Fig).

We suggest a possible model for a gliding mechanism that integrates all information to date (Fig 6C). Movements for gliding may be generated in the bowl complex and transmitted to the paired plates fixed to the cell front through the terminal button. Then, extension and retraction of the organelle repeat. The P1 adhesin complexes connected to the internal core repeat a catch-pull-release cycle with sialylated oligosaccharides on the host surface. The organelle pulls the other parts of the cell connected to the back end of the bowl complex.

## Materials and Methods

### Strains and culture

The bacterial strains and plasmids are listed in S2 Table. *M*. *pneumoniae* strain M129 (the type strain of subtype 1) [55] was used as the wild-type strain and the recipient of transposons. *M*. *pneumoniae* cells were grown in Aluotto or PPLO medium at 37°C in a tissue culture flask [7, 18]. *Escherichia coli* strains DB3.1, DH5α, and JM83 were used for plasmid construction. For the selection and maintenance of antibiotic-resistant strains of *M*. *pneumoniae* and *E*. *coli*, antibiotics were used at the following concentrations: gentamicin, 18 μg/ml; chloramphenicol, 15 μg/ml; ampicillin, 50 μg/ml; kanamycin, 50 μg/ml.

### Isolation and identification of the core fraction

In a tissue culture flask, *M*. *pneumoniae* cells from 0.5 L culture in the exponential phase were washed three times with phosphate-buffered saline (PBS) consisting of 75 mM sodium phosphate (pH 7.3) and 68 mM NaCl. The cells attached to the bottom of the flask were suspended to be a calculated optical density at 600 nm of 20 in PBS containing 0.1 mM phenylmethylsulfonyl fluoride (PMSF), and then 1% (vol/vol) Triton X-100 and 0.1 mg/ml DNase I from bovine pancreas (Sigma) were added. After gentle shaking for 5 min at RT, the lysate was put on the top of the sucrose layers, subjected to a stepwise density gradient consisting of 0%, 20%, 30%, 40%, 50%, and 60% sucrose layers in PBS, and centrifuged at 25,000 × g for 20 min in a 1.5 ml microtube rotor, MX-100 (TOMY, Tokyo, Japan). A fraction of 0.2 ml was recovered from the bottom of layer 40% as the core fraction. Using TCA precipitation, the layers of 0%, 20%, and 30% sucrose were combined and the proteins were recovered as a soluble fraction. The whole cell suspension, soluble fraction, and core fraction were subjected to SDS-PAGE and the protein bands were identified by peptide mass fingerprinting (PMF), as described previously [56].

### Electron microscopy

The cell suspension and core fraction were placed directly onto carbon-coated, glow-discharged grids for 5 min at RT. The grids were treated by 0.01–1% Triton X-100 or 1% Tween 20, 1 mg/ml DNase, and 5 mM MgCl_2_ in PBS for 30 s at RT. After the removal of the solution, the grid was washed with PBS, stained by 2% ammonium molybdate (vol/vol), air-dried, and observed by EM as previously described [6, 56]. For immuno-gold EM, the culture of *M*. *pneumoniae* cells was put onto a grid and incubated for 5 min at RT [57]. After the liquid was removed, the grid was treated with 1% Triton X-100 and 0.1 mg/ml DNase in PBS for 1 min. Cells were labeled with 1/100 diluted antiserum against GFP (Thermo Fisher Scientific, Waltham, MA, USA) in PBS containing 2% BSA (PBS-B) as the primary antibody, and washed five times by PBS. Subsequently, the grids were treated with 1/10 diluted 5 nm colloidal-gold-labeled goat antibody (Sigma) in PBS-B for 30 min at RT as the secondary antibody, washed five times by PBS, and then stained negatively with the ammonium molybdate.

### Light microscopy

*M*. *pneumoniae* cells expressing EYFP-fused protein were observed under an inverted microscope, IX71 (Olympus, Tokyo, Japan). Images were recorded with a CCD (charge-coupled device) camera ORCA-R2 (Hamamatsu Photonics, Hamamatsu, Japan) and DP30BW (Olympus). A culture of 2 ml was grown in a glass-bottom dish (Iwaki, Tokyo, Japan) and observed directly. Immunofluorescence microscopy was done using the anti-P1 antibodies, as previously reported [19, 31, 32]. As a standard of image density, carboxylated polystyrene beads of 200 nm diameter (Polysciences, Warrington, PA, USA) were used. The beads were suspended in PBS containing 2 mM MgCl_2_ at 1/1000 the concentration of the original stock, put on the glass after the cell culture was removed, and kept for 5 min at RT. The cells and beads were washed three times with PBS containing MgCl_2_ before observation. Image data were acquired by AQUACOSMOS software (Hamamatsu Photonics) and analyzed by Image J 1.45s (http://rsb.info.nih.gov/ij/) and IGOR Pro ver 6.2 (WaveMetrics, Lake Oswego, OR, USA).

## Supporting Information

S1 Fig Variations in EM images of internal cores. Each internal core can be divided into three parts: a terminal button, paired plates, and a bowl (wheel) complex. Yellow arrows indicate the boundaries between parts. Bold (A), slim (B), fork (C), and branched (D) types are aligned as the cell front on the right. A less-dense area in (C) is marked by an asterisk.
(TIF)

S2 Fig Protein localization examined by fluorescence in living cells. (A) Single-protein labeling. The ORFs fused with the *eyfp* gene at the 5’ or 3’ terminus (marked only for the C terminus beneath the ORF code) were expressed under the *tuf* promoter. P1 adhesin was labeled also red by a monoclonal antibody. Twenty-nine proteins were fused with EYFP and expressed in the genetic background of the wild type. The schematic is the localization pattern of fluorescence signals in living cells. The patterns were classified into four types: in type "a", a focused signal is observed at the attachment organelle; in type "f", a focal localization; in type "d", diffuse signal in the whole cell; in type "n", no signals. The fluorescence localization pattern is shown in the upper right of each panel. Images are assigned according to the categories shown in Fig 3. The Gene ID is presented in the upper left of each panel with its annotation. Fluorescence and phase-contrast images are merged. (B) Double-protein labeling. The ORFs fused with *eyfp* and *ecfp* were expressed under the *tuf* promoter.
(TIF)

S3 Fig Heterogeneity of fusion proteins of 11 constructs. (A) Fluorescence signals in a field 40 μm wide and 27 μm high. Fluorescence and phase-contrast images are merged. (Upper) Individual proteins were labeled. (Lower) HMW2 labeled by EYFP at the N terminus and HMW2 labeled by CYFP at the C terminus were expressed and detected individually (middle and right), and merged (left). (B) Electrophoretic analysis for 11 constructs. (Upper) SDS-10% to 20% gradient PAGE image stained by CBB. (Middle) Western blotting detecting EYFP. The major bands are marked by a red triangle. Size standards are shown in the left. The constructs examined are listed in the lower table with molecular weights based on the amino acid sequences and band positions.
(TIF)

S4 Fig Fluorescence and image-density profiles of cell labeled for organelle protein. A typical cell image is shown for phase-contrast (left), fluorescence (center), and merged (right) images are shown on the upper of each panel. The profiles of fluorescence and image density are shown by colored and gray lines as relative values in the graph on the lower of each panel. The subcellular positions along the cell axis and image density were determined as shown in S5 Fig.
(TIF)

S5 Fig Mapping procedure of fluorescent foci on the attachment organelle. (A) EYFP fused to P30 (MPN453) in a living cell. Phase-contrast, fluorescence, and merged images of the cell. A 200-nm bead is attached to the glass surface at the lower left in the field. (B) Profiling image densities of bead (broken), cell (solid), and EYFP signal (green), along the lines shown on the images in (A). The image density of the bead at its peak position and the averaged image density of the glass surface were defined as 0% and 100%, respectively. The position on the axis at 16 ± 5% image density in the phase-contrast image is defined as the cell edge, which corresponds to the peak position of P30-EYFP fluorescence. (C) Variations in image densities of bead (upper left), cell (upper right), and EYFP signal (lower right). The image densities were profiled in the same way with (B) for 10 images, and overlaid with different colors.
(TIF)

S6 Fig Protein clusters based on subcellular localizing positions. The protein pairs from different clusters were supported for significant positional difference by less than 0.05 *p*-value of ANOVA test for single and double fluorescence labeling as listed in S3 Table. ✝, ‡: TopJ—P200 and HMW3—HMW2 pairs were not supported partly for significant difference by the test.
(TIF)

S1 Table Summary of proteins found in core fraction and previously reported components of attachment organelle. (DOCX)

S2 Table Plasmids used in this study. (DOCX)

S3 Table *p*-values of ANOVA test for all pairs of organelle protein positions and distances. (Upper) Tests for signal positions obtained from single labeling. (Lower) Tests for distances obtained from double labeling. The significant difference between a pair of distances including the same protein may support the positional difference between the other two proteins. The p-values less than 0.05 showing significant difference are colored red.
(DOCX)

## References

[1] Razin S, Yogev D, Naot Y. Molecular biology and pathogenicity of mycoplasmas. Microbiol Mol Biol Rev. 1998;62(4):1094–156. . 9841667 10.1128/mmbr.62.4.1094-1156.1998 PMC98941

[2] Yamada M, Buller R, Bledsoe S, Storch GA. Rising rates of macrolide-resistant Mycoplasma pneumoniae in the central United States. Pediatr Infect Dis J. 2012;31(4):409–0. 10.1097/INF.0b013e318247f3e0 22209916

[3] Bebear C, Pereyre S, Peuchant O. Mycoplasma pneumoniae: susceptibility and resistance to antibiotics. Future Microbiol. 2011;6(4):423–31. 10.2217/fmb.11.18 21526943

[4] Okazaki N, Narita M, Yamada S, Izumikawa K, Umetsu M, Kenri T, et al Characteristics of macrolide-resistant *Mycoplasma pneumoniae* strains isolated from patients and induced with erythromycin in vitro. Microbiol Immunol. 2001;45(8):617–20. . 11592636 10.1111/j.1348-0421.2001.tb01293.x

[5] Miyata M, Nakane D. Gliding mechanism of *Mycoplasma pneumoniae* subgroup implication from *Mycoplasma mobile* In: Browning G, Citti C, editors. Molecular and Cell Biology of *Mollicutes*. Norfolk, UK: Horizon Press; 2013 p. 237–52.

[6] Nakane D, Miyata M. Cytoskeletal asymmetrical-dumbbell structure of a gliding mycoplasma, *Mycoplasma gallisepticum*, revealed by negative-staining electron microscopy. J Bacteriol. 2009;191(10):3256–64. 10.1128/JB.01823-08 19286806 PMC2687163

[7] Kenri T, Seto S, Horino A, Sasaki Y, Sasaki T, Miyata M. Use of fluorescent-protein tagging to determine the subcellular localization of *Mycoplasma pneumoniae* proteins encoded by the cytadherence regulatory locus. J Bacteriol. 2004;186(20):6944–55. . 15466048 10.1128/JB.186.20.6944-6955.2004 PMC522203

[8] Radestock U, Bredt W. Motility of *Mycoplasma pneumoniae* . J Bacteriol. 1977;129(3):1495–501. . 14925 10.1128/jb.129.3.1495-1501.1977 PMC235127

[9] Prince OA, Krunkosky TM, Krause DC. In vitro spatial and temporal analysis of *Mycoplasma pneumoniae* colonization of human airway epithelium. Infect Immun. 2014;82(2):579–86. 10.1128/IAI.01036-13 24478073 PMC3911394

[10] Miyata M. Unique centipede mechanism of *Mycoplasma* gliding. Annu Rev Microbiol. 2010;64:519–37. 10.1146/annurev.micro.112408.134116 20533876

[11] Miyata M. Centipede and inchworm models to explain *Mycoplasma* gliding. Trends Microbiol. 2008;16(1):6–12. . 18083032 10.1016/j.tim.2007.11.002

[12] Krause DC, Balish MF. Cellular engineering in a minimal microbe: structure and assembly of the terminal organelle of *Mycoplasma pneumoniae* . Mol Microbiol. 2004;51:917–24. . 14763969 10.1046/j.1365-2958.2003.03899.x

[13] Dandekar T, Huynen M, Regula JT, Ueberle B, Zimmermann CU, Andrade MA, et al Re-annotating the *Mycoplasma pneumoniae* genome sequence: adding value, function and reading frames. Nucleic Acids Res. 2000;28(17):3278–88. . 10954595 10.1093/nar/28.17.3278 PMC110705

[14] Krause DC. *Mycoplasma pneumoniae* cytadherence: unravelling the tie that binds. Mol Microbiol. 1996;20:247–53. . 8733224 10.1111/j.1365-2958.1996.tb02613.x

[15] Seybert A, Herrmann R, Frangakis AS. Structural analysis of *Mycoplasma pneumoniae* by cryo-electron tomography. J Struct Biol. 2006;156(2):342–54. . 16875842 10.1016/j.jsb.2006.04.010

[16] Henderson GP, Jensen GJ. Three-dimensional structure of *Mycoplasma pneumoniae*'s attachment organelle and a model for its role in gliding motility. Mol Microbiol. 2006;60(2):376–85. . 16573687 10.1111/j.1365-2958.2006.05113.x

[17] Kasai T, Nakane D, Ishida H, Ando H, Kiso M, Miyata M. Role of binding in *Mycoplasma mobile* and *Mycoplasma pneumoniae* gliding analyzed through inhibition by synthesized sialylated compounds. J Bacteriol. 2012;195(3):429–35. 10.1128/JB.01141-12 23123913 PMC3554017

[18] Nakane D, Adan-Kubo J, Kenri T, Miyata M. Isolation and characterization of P1 adhesin, a leg protein of the gliding bacterium *Mycoplasma pneumoniae* . J Bacteriol. 2011;193:715–22. 10.1128/JB.00796-10 21097617 PMC3021217

[19] Seto S, Kenri T, Tomiyama T, Miyata M. Involvement of P1 adhesin in gliding motility of *Mycoplasma pneumoniae* as revealed by the inhibitory effects of antibody under optimized gliding conditions. J Bacteriol. 2005;187(5):1875–7. 15716461 10.1128/JB.187.5.1875-1877.2005 PMC1064011

[20] Gobel U, Speth V, Bredt W. Filamentous structures in adherent *Mycoplasma pneumoniae* cells treated with nonionic detergents. J Cell Biol. 1981;91:537–43. . 6796593 10.1083/jcb.91.2.537 PMC2111982

[21] Meng KE, Pfister RM. Intracellular structures of *Mycoplasma pneumoniae* revealed after membrane removal. J Bacteriol. 1980;144:390–9. . 6774963 10.1128/jb.144.1.390-399.1980 PMC294663

[22] Hegermann J, Herrmann R, Mayer F. Cytoskeletal elements in the bacterium *Mycoplasma pneumoniae* . Naturwissenschaften. 2002;89(10):453–8. . 12384719 10.1007/s00114-002-0359-2

[23] Regula JT, Boguth G, Gorg A, Hegermann J, Mayer F, Frank R, et al Defining the mycoplasma 'cytoskeleton': the protein composition of the Triton X-100 insoluble fraction of the bacterium Mycoplasma pneumoniae determined by 2-D gel electrophoresis and mass spectrometry. Microbiology. 2001;147(Pt 4):1045–57. . 11283300 10.1099/00221287-147-4-1045

[24] Balish MF. *Mycoplasma pneumoniae*, an underutilized model for bacterial cell biology. J Bacteriol. 2014;196(21):3675–82. 10.1128/JB.01865-14 25157081 PMC4248795

[25] Kahn R, Rubin RW. Quantitation of submicrogram amounts of protein using coomassie brilliant blue R on sodium dodecyl sulfate-polyacrylamide slab-gels. Anal Biochem. 1975;67(1):347–52. . 50023 10.1016/0003-2697(75)90305-x PMC8334758

[26] Balish M. Organization of the cyotoskeletons of diverse *Mollicutes* In: Browning G, Citti C, editors. Molecular and Cell Biology of *Mollicutes*. Norfolk, UK: Horizon Press; 2013 p. 237–52.

[27] Kuhner S, van Noort V, Betts MJ, Leo-Macias A, Batisse C, Rode M, et al Proteome organization in a genome-reduced bacterium. Science. 2009;326(5957):1235–40. 10.1126/science.1176343 19965468

[28] Tulum I, Yabe M, Uenoyama A, Miyata M. Localization of P42 and F1-ATPase alpha-subunit homolog of the gliding machinery in *Mycoplasma mobile* revealed by newly developed gene manipulation and fluorescent protein tagging. J Bacteriol. 2014;196(10):1815–24. 10.1128/JB.01418-13 24509320 PMC4011001

[29] Knudtson KL, Minion FC. Construction of Tn*4001lac* derivatives to be used as promoter probe vectors in mycoplasmas. Gene. 1993;137(2):217–22. . 8299950 10.1016/0378-1119(93)90009-r

[30] Hasselbring BM, Jordan JL, Krause RW, Krause DC. Terminal organelle development in the cell wall-less bacterium *Mycoplasma pneumoniae* . Proc Natl Acad Sci USA. 2006;103(44):16478–83. . 17062751 10.1073/pnas.0608051103 PMC1637607

[31] Seto S, Miyata M. Attachment organelle formation represented by localization of cytadherence protein and formation of electron-dense core in the wild-type and mutant strains of *Mycoplasma pneumoniae* . J Bacteriol. 2003;185:1082–91. . 12533484 10.1128/JB.185.3.1082-1091.2003 PMC142798

[32] Seto S, Layh-Schmitt G, Kenri T, Miyata M. Visualization of the attachment organelle and cytadherence proteins of *Mycoplasma pneumoniae* by immunofluorescence microscopy. J Bacteriol. 2001;183:1621–30. . 11160093 10.1128/JB.183.5.1621-1630.2001 PMC95047

[33] Hasselbring BM, Sheppard ES, Krause DC. P65 truncation impacts P30 dynamics during *Mycoplasma pneumoniae* gliding. J Bacteriol. 2012;194(11):3000–7. 10.1128/JB.00091-12 22544269 PMC3370611

[34] Kenri T, Seto S, Horino A, Sasaki T, Miyata M, editors. Mapping of localization sites of cytadherence-related and cytoskeletal proteins of *Mycoplasma pneumoniae* by fluorescent protein tagging The 16th meeting of the International Organization for Mycoplasmology; 2006; Cambridge, UK.

[35] Layh-Schmitt G, Herrmann R. Localization and biochemical characterization of the ORF6 gene product of the *Mycoplasma pneumoniae* P1 operon. Infect Immun. 1992;60(7):2906–13. . 1612757 10.1128/iai.60.7.2906-2913.1992 PMC257253

[36] Romero-Arroyo CE, Jordan J, Peacock SJ, Willby MJ, Farmer MA, Krause DC. *Mycoplasma pneumoniae* protein P30 is required for cytadherence and associated with proper cell development. J Bacteriol. 1999;181(4):1079–87. . 9973332 10.1128/jb.181.4.1079-1087.1999 PMC93483

[37] Krause DC, Proft T, Hedreyda CT, Hilbert H, Plagens H, Herrmann R. Transposon mutagenesis reinforces the correlation between *Mycoplasma pneumoniae* cytoskeletal protein HMW2 and cytadherence. J Bacteriol. 1997;179(8):2668–77. . 9098066 10.1128/jb.179.8.2668-2677.1997 PMC179017

[38] Balish MF, Santurri RT, Ricci AM, Lee KK, Krause DC. Localization of *Mycoplasma pneumoniae* cytadherence-associated protein HMW2 by fusion with green fluorescent protein: implications for attachment organelle structure. Mol Microbiol. 2003;47:49–60. . 12492853 10.1046/j.1365-2958.2003.03282.x

[39] Bose SR, Balish MF, Krause DC. *Mycoplasma pneumoniae* cytoskeletal protein HMW2 and the architecture of the terminal organelle. J Bacteriol. 2009;191(21):6741–8. 10.1128/JB.01486-08 19717588 PMC2795293

[40] Boonmee A, Ruppert T, Herrmann R. The gene mpn310 (hmw2) from Mycoplasma pneumoniae encodes two proteins, HMW2 and HMW2-s, which differ in size but use the same reading frame. FEMS Microbiol Lett. 2009;290(2):174–81. 10.1111/j.1574-6968.2008.01422.x 19025563

[41] Fisseha M, Gohlmann HW, Herrmann R, Krause DC. Identification and complementation of frameshift mutations associated with loss of cytadherence in *Mycoplasma pneumoniae* . J Bacteriol. 1999;181(14):4404–10. . 10400600 10.1128/jb.181.14.4404-4410.1999 PMC93944

[42] Papazisi L, Gorton TS, Kutish G, Markham PF, Browning GF, Nguyen DK, et al The complete genome sequence of the avian pathogen Mycoplasma gallisepticum strain R(low). Microbiology. 2003;149(Pt 9):2307–16. . 12949158 10.1099/mic.0.26427-0

[43] Fraser CM, Gocayne JD, White O, Adams MD, Clayton RA, Fleischmann RD, et al The minimal gene complement of *Mycoplasma genitalium* . Science. 1995;270(5235):397–403. . 7569993 10.1126/science.270.5235.397

[44] Hasselbring BM, Page CA, Sheppard ES, Krause DC. Transposon mutagenesis identifies genes associated with *Mycoplasma pneumoniae* gliding motility. J Bacteriol. 2006;188(17):6335–45. . 16923901 10.1128/JB.00698-06 PMC1595379

[45] Jordan JL, Chang HY, Balish MF, Holt LS, Bose SR, Hasselbring BM, et al Protein P200 is dispensable for *Mycoplasma pneumoniae* hemadsorption but not gliding motility or colonization of differentiated bronchial epithelium. Infect Immun. 2007;75:518–22. . 17043103 10.1128/IAI.01344-06 PMC1828431

[46] Stevenson G, Lee SJ, Romana LK, Reeves PR. The cps gene cluster of Salmonella strain LT2 includes a second mannose pathway: sequence of two genes and relationship to genes in the *rfb* gene cluster. Mol Gen Genet. 1991;227(2):173–80. . 1712067 10.1007/BF00259668

[47] Jenal U. The role of proteolysis in the *Caulobacter crescentus* cell cycle and development. Res Microbiol. 2009;160(9):687–95. 10.1016/j.resmic.2009.09.006 19781638

[48] Tsilibaris V, Maenhaut-Michel G, Van Melderen L. Biological roles of the Lon ATP-dependent protease. Res Microbiol. 2006;157(8):701–13. . 16854568 10.1016/j.resmic.2006.05.004

[49] Balish MF, Hahn TW, Popham PL, Krause DC. Stability of *Mycoplasma pneumoniae* cytadherence-accessory protein HMW1 correlates with its association with the triton shell. J Bacteriol. 2001;183(12):3680–8. . 11371532 10.1128/JB.183.12.3680-3688.2001 PMC95245

[50] Cloward JM, Krause DC. *Mycoplasma pneumoniae* J-domain protein required for terminal organelle function. Mol Microbiol. 2009;71(5):1296–307. 10.1111/j.1365-2958.2009.06602.x 19183275 PMC5833977

[51] Hasselbring BM, Krause DC. Cytoskeletal protein P41 is required to anchor the terminal organelle of the wall-less prokaryote *Mycoplasma pneumoniae* . Mol Microbiol. 2007;63(1):44–53. . 17163973 10.1111/j.1365-2958.2006.05507.x

[52] Hu PC, Cole RM, Huang YS, Graham JA, Gardner DE, Collier AM, et al *Mycoplasma pneumoniae* infection: role of a surface protein in the attachment organelle. Science. 1982;216:313–5. . 6801766 10.1126/science.6801766

[53] Feldner J, Gobel U, Bredt W. *Mycoplasma pneumoniae* adhesin localized to tip structure by monoclonal antibody. Nature. 1982;298:765–7. . 7110314 10.1038/298765a0

[54] Baseman JB, Cole RM, Krause DC, Leith DK. Molecular basis for cytadsorption of *Mycoplasma pneumoniae* . J Bacteriol. 1982;151:1514–22. . 6809731 10.1128/jb.151.3.1514-1522.1982 PMC220433

[55] Lipman RP, Clyde WA Jr., Denny FW. Characteristics of virulent, attenuated, and avirulent *Mycoplasma pneumoniae* strains. J Bacteriol. 1969;100(2):1037–43. . 5359607 10.1128/jb.100.2.1037-1043.1969 PMC250191

[56] Nakane D, Miyata M. Cytoskeletal “Jellyfish” structure of Mycoplasma mobile. Proc Natl Acad Sci USA. 2007;104:19518–23. . 18042728 10.1073/pnas.0704280104 PMC2148321

[57] Nakane D, Miyata M. *Mycoplasma mobile* cells elongated by detergent and their pivoting movements in gliding. J Bacteriol. 2012;194(1):122–30. 10.1128/JB.05857-11 22001513 PMC3256606

