# Supplementary figures and images for: Systematic Structural Analyses of Attachment Organelle in Mycoplasma pneumoniae
Source: PLoS Pathog. 2015 Dec 3;11(12):e1005299. doi: 10.1371/journal.ppat.1005299 (PMC4669176; doi:10.1371/journal.ppat.1005299)

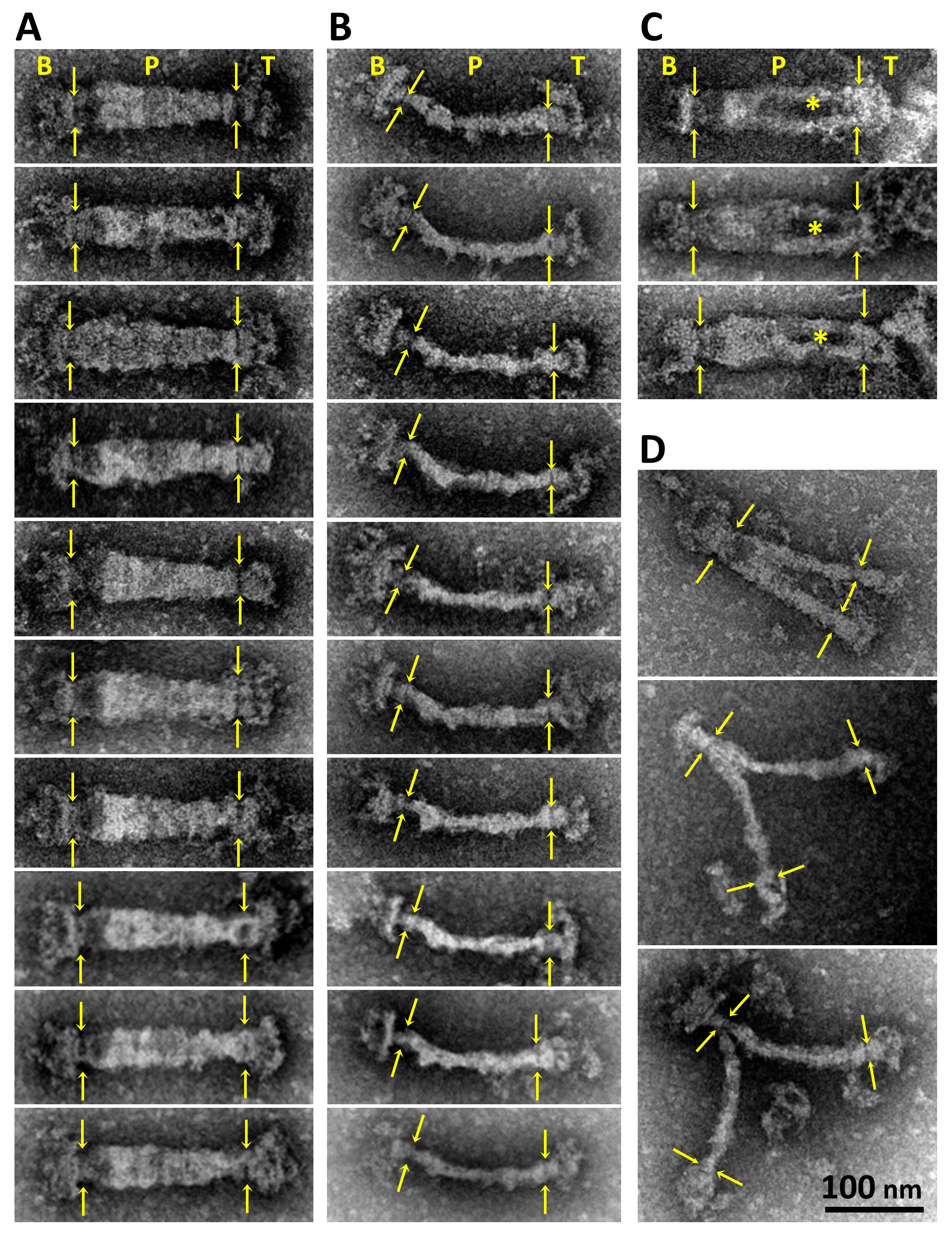

Supplement: S1 Fig — Each internal core can be divided into three parts: a terminal button, paired plates, and a bowl (wheel) complex. Yellow arrows indicate the boundaries between parts. Bold (A), slim (B), fork (C), and branched (D) types are aligned as the cell front on the right. A less-dense area in (C) is marked by an asterisk. (TIF) [file ppat.1005299.s001.tif]

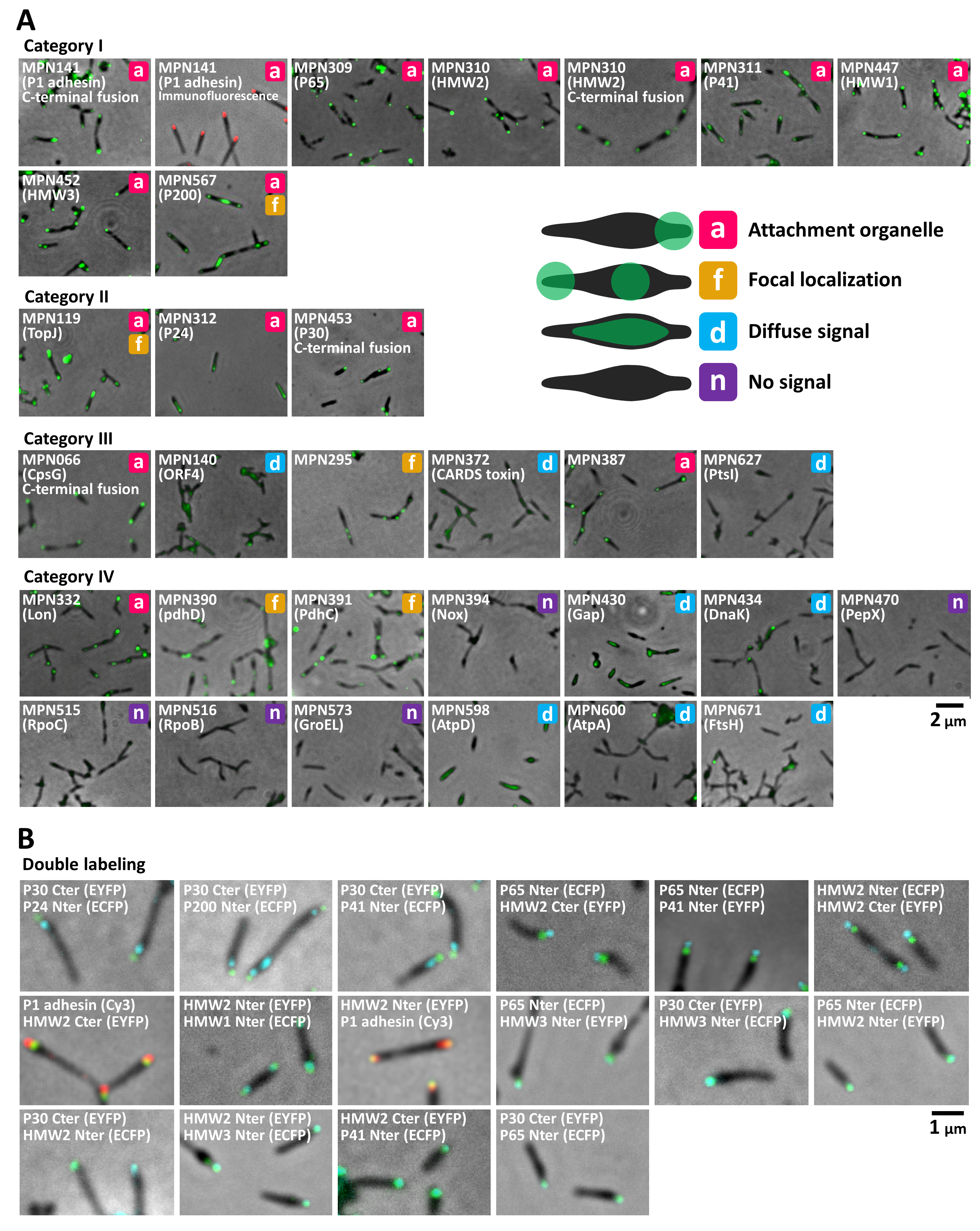

Supplement: S2 Fig — (A) Single-protein labeling. The ORFs fused with the eyfp gene at the 5’ or 3’ terminus (marked only for the C terminus beneath the ORF code) were expressed under the tuf promoter. P1 adhesin was labeled also red by a monoclonal antibody. Twenty-nine proteins were fused with EYFP and expressed in the genetic background of the wild type. The schematic is the localization pattern of fluorescence signals in living cells. The patterns were classified into four types: in type "a", a focused signal is observed at the attachment organelle; in type "f", a focal localization; in type "d", diffuse signal in the whole cell; in type "n", no signals. The fluorescence localization pattern is shown in the upper right of each panel. Images are assigned according to the categories shown in Fig 3. The Gene ID is presented in the upper left of each panel with its annotation. Fluorescence and phase-contrast images are merged. (B) Double-protein labeling. The ORFs fused with eyfp and ecfp were expressed under the tuf promoter. (TIF) [file ppat.1005299.s002.tif]

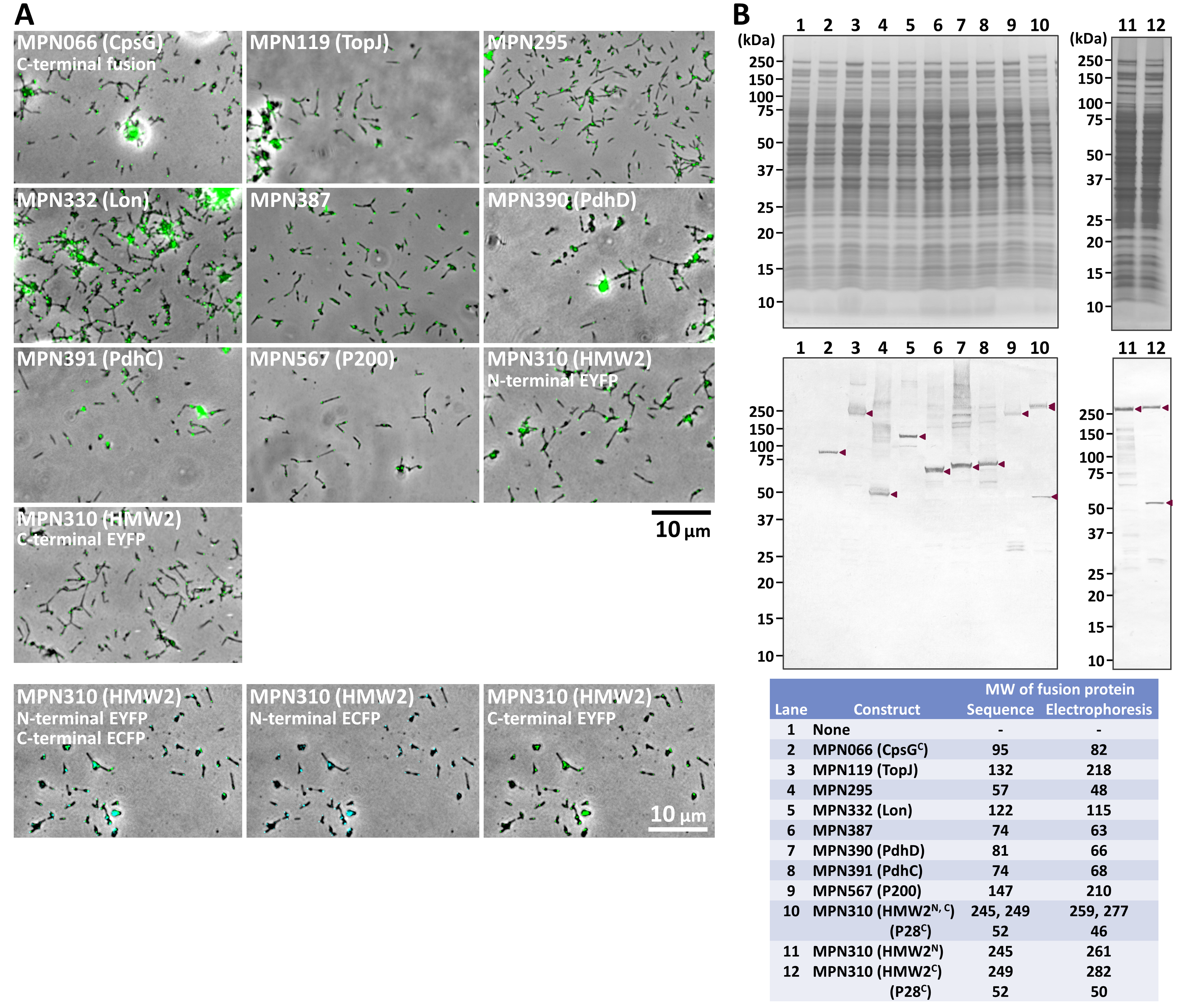

Supplement: S3 Fig — (A) Fluorescence signals in a field 40 μm wide and 27 μm high. Fluorescence and phase-contrast images are merged. (Upper) Individual proteins were labeled. (Lower) HMW2 labeled by EYFP at the N terminus and HMW2 labeled by CYFP at the C terminus were expressed and detected individually (middle and right), and merged (left). (B) Electrophoretic analysis for 11 constructs. (Upper) SDS-10% to 20% gradient PAGE image stained by CBB. (Middle) Western blotting detecting EYFP. The major bands are marked by a red triangle. Size standards are shown in the left. The constructs examined are listed in the lower table with molecular weights based on the amino acid sequences and band positions. (TIF) [file ppat.1005299.s003.tif]

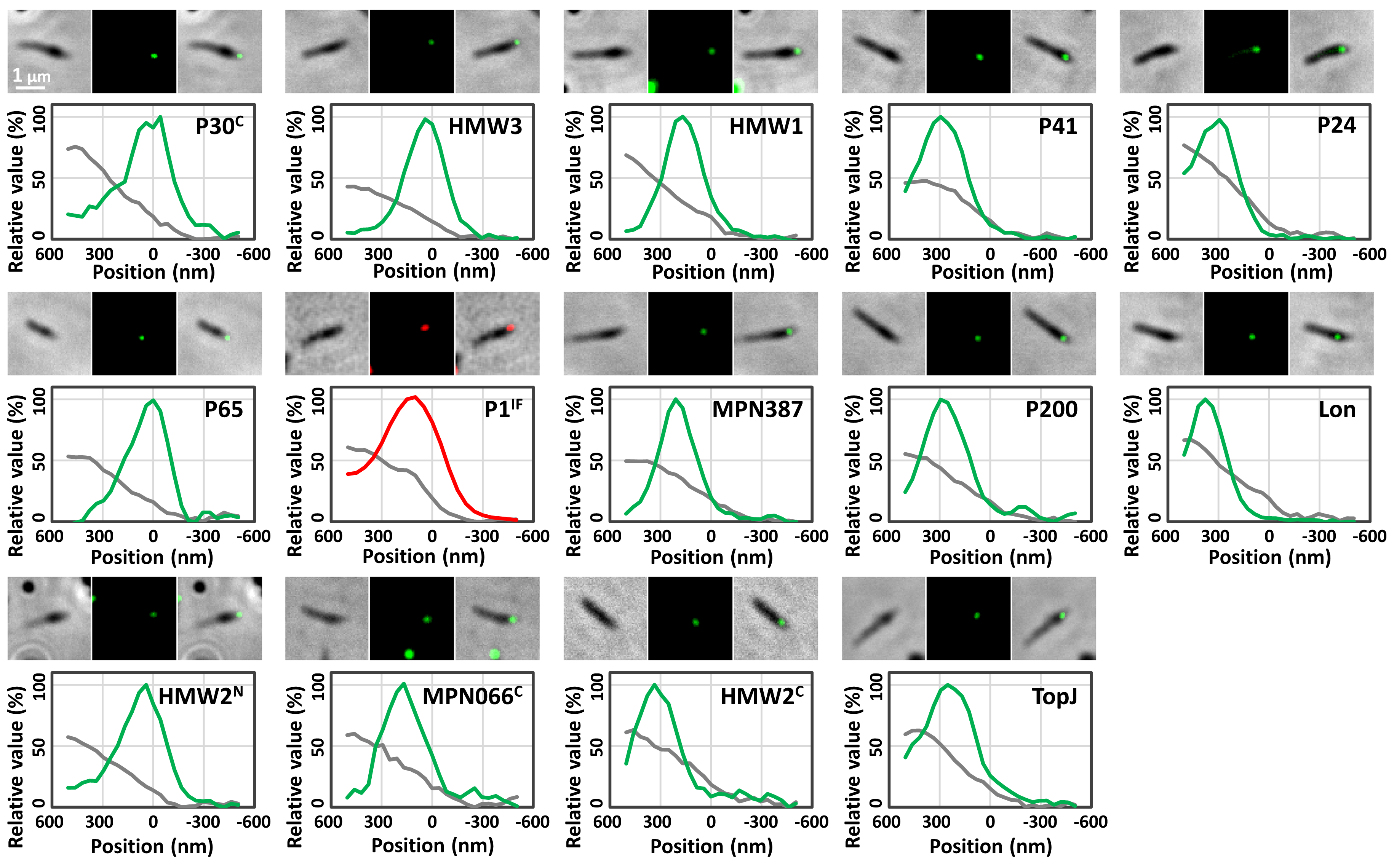

Supplement: S4 Fig — A typical cell image is shown for phase-contrast (left), fluorescence (center), and merged (right) images are shown on the upper of each panel. The profiles of fluorescence and image density are shown by colored and gray lines as relative values in the graph on the lower of each panel. The subcellular positions along the cell axis and image density were determined as shown in S5 Fig. (TIF) [file ppat.1005299.s004.tif]

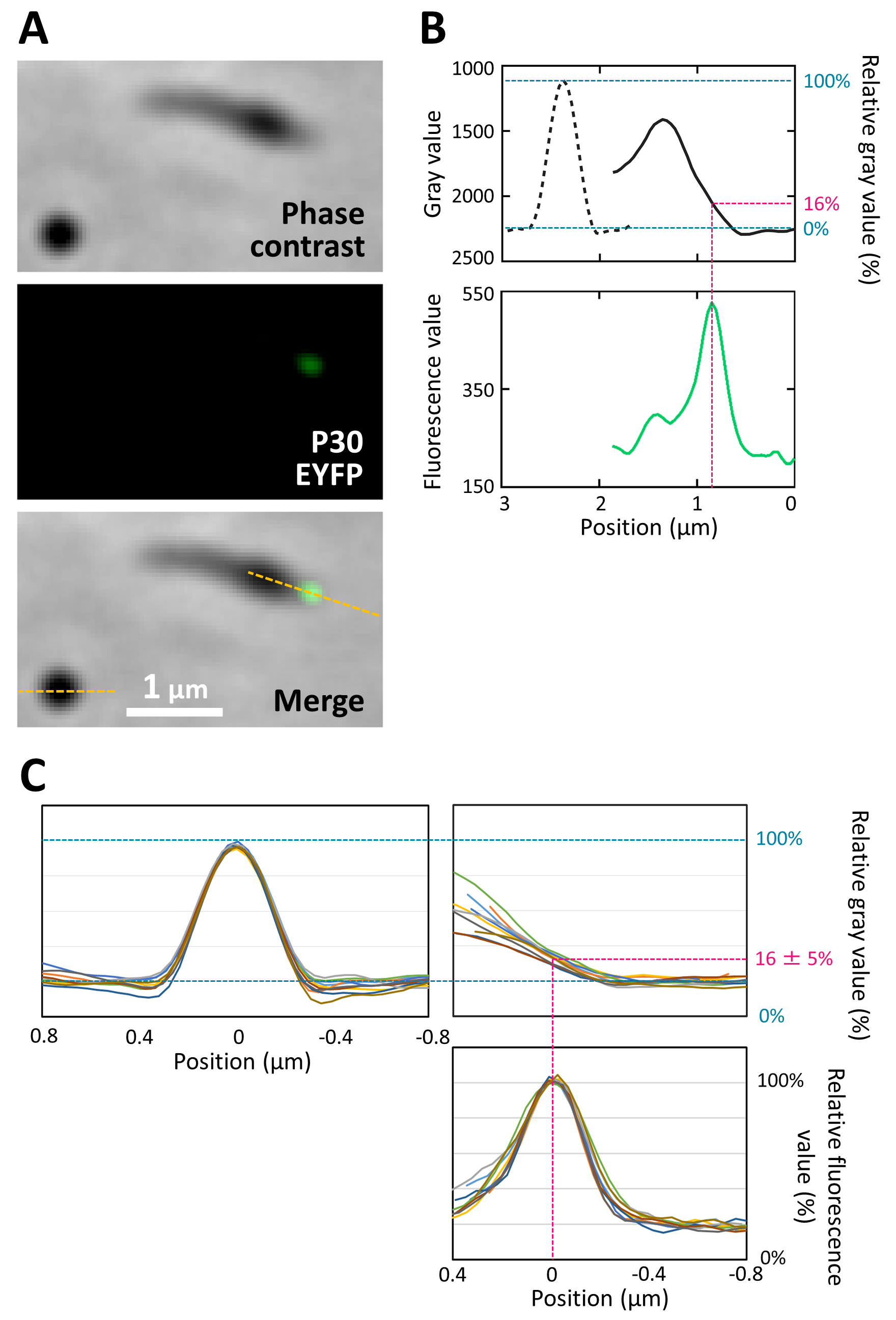

Supplement: S5 Fig — (A) EYFP fused to P30 (MPN453) in a living cell. Phase-contrast, fluorescence, and merged images of the cell. A 200-nm bead is attached to the glass surface at the lower left in the field. (B) Profiling image densities of bead (broken), cell (solid), and EYFP signal (green), along the lines shown on the images in (A). The image density of the bead at its peak position and the averaged image density of the glass surface were defined as 0% and 100%, respectively. The position on the axis at 16 ± 5% image density in the phase-contrast image is defined as the cell edge, which corresponds to the peak position of P30-EYFP fluorescence. (C) Variations in image densities of bead (upper left), cell (upper right), and EYFP signal (lower right). The image densities were profiled in the same way with (B) for 10 images, and overlaid with different colors. (TIF) [file ppat.1005299.s005.tif]

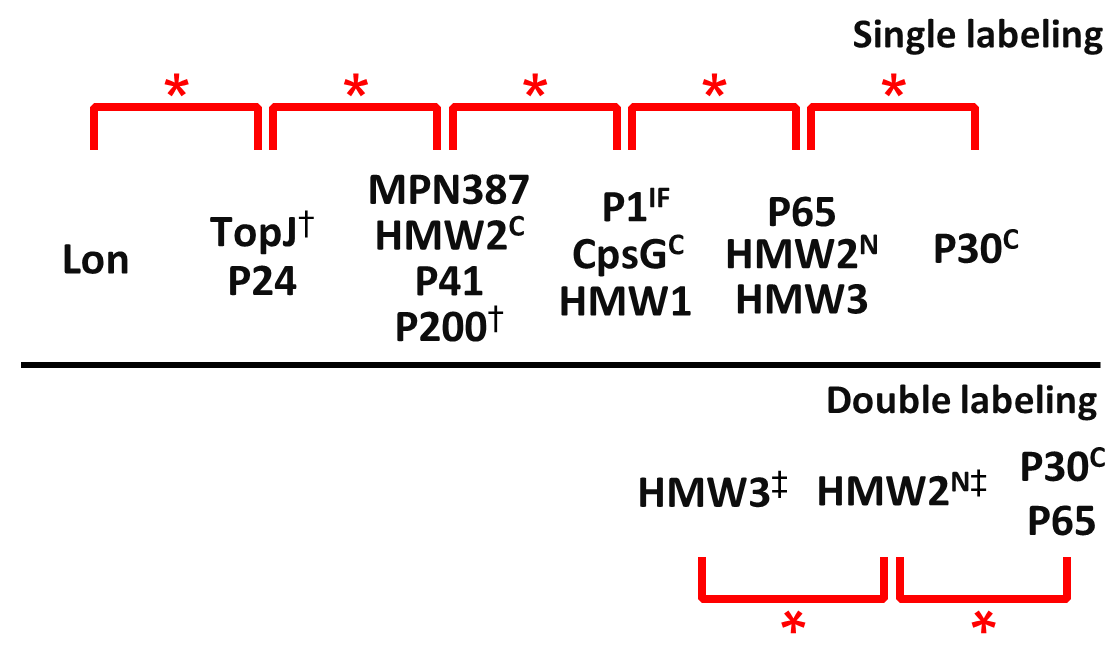

Supplement: S6 Fig — The protein pairs from different clusters were supported for significant positional difference by less than 0.05 p-value of ANOVA test for single and double fluorescence labeling as listed in S3 Table. ✝, ‡: TopJ—P200 and HMW3—HMW2 pairs were not supported partly for significant difference by the test. (TIF) [file ppat.1005299.s006.tif]
