# Supplementary material for: Systematic Structural Analyses of Attachment Organelle in Mycoplasma pneumoniae
Source: PLoS Pathog. 2015 Dec 3;11(12):e1005299. doi: 10.1371/journal.ppat.1005299 (PMC4669176; doi:10.1371/journal.ppat.1005299)
Supplement: S1 Table — (DOCX) [file ppat.1005299.s007.docx]

**Table S1** Summary of proteins found in core fraction and previously reported components of attachment organelle.

|  |  |  |  | **Amino acid number** | **Core-rich specific^§^** | **MALDI-TOF^‡^** | | **EYFP  signal^¶^** | **Molar ratio^†^** |
| --- | --- | --- | --- | --- | --- | --- | --- | --- | --- |
| **Category^*^** |  | **Gene ID** | **Protein name and annotation** |  |  | **Exp.** | **Cov.** |  |  |
| I |  | MPN141 | P1 adhesin | 1627 | N | 1.1×10^-14^ | 27% | d^N^, a^C^, a^IF^ | 58 |
|  |  | MPN142a | P40 (Protein C) | 454 | N | 2.0×10^-2^ | 7% | d^N^, a^IF^ | 151 |
|  |  | MPN142b | P90 (Protein B) | 764 | N | 7.1×10^-12^ | 16% | d^N^, n^C^, a^IF^ | 74 |
|  |  | MPN309 | P65 | 405 | S | 3.5×10^-2^ | 13% | a^N^ | 71 |
|  |  | MPN310 | HMW2 | 1818 | S | 2.7×10^-12^ | 23% | a^N^, a^C^ | 100 |
|  |  | MPN311 | P41 | 357 | S | 6.1×10^-8^ | 36% | a^N^ | 162 |
|  |  | MPN447 | HMW1 | 1018 | S | 3.2×10^-18^ | 23% | a^N^ | 55 |
|  |  | MPN452 | HMW3 | 672 | S | 1.2×10^-17^ | 37% | a^N^ | 96 |
|  |  | MPN567 | P200 | 1036 | S | 1.9×10^-4^ | 6% | a or f^N^ | 16 |
| II |  | MPN119 | TopJ | 910 | ND | - | - | a or f^N^ | - |
|  |  | MPN312 | P24 | 218 | ND | - | - | a^N^ | - |
|  |  | MPN453 | P30 | 274 | ND | - | - | d^N^, a^C^ | - |
| III | III-a | MPN066 | CpsG: Phosphomannomutase | 554 | S | 4.1×10^-3^ | 8% | d^N^, a^C^ | 73 |
|  |  | MPN387 | - | 358 | S | 1.3×10^-5^ | 27% | a^N^ | 94 |
|  |  | MPN140 | DHH phosphoesterase, ORF4 | 324 | S | 3.0×10^-6^ | 33% | d^N^, f^C^ | - |
|  |  | MPN295 | - | 220 | S | 7.5×10^-2^ | 34% | f^N^, f^C^ | - |
|  |  | MPN372 | CARDS toxin: Pertussis toxin subunit 1 | 591 | S | 3.0×10^-16^ | 37% | d^N^, d^C^ | - |
|  |  | MPN627 | PtsI: Phosphotransferase system, enzyme I | 572 | S | 6.8×10^-4^ | 20% | d^N^, n^C^ | - |
| IV | IV-a | MPN332 | Lon: ATP-dependent protease Lon | 797 | N | 3.4×10^-10^ | 23% | a^N^ | 62 |
|  |  | MPN207 | PtsG: PTS system, glucose-specific IIABC | 940 | N | 4.2×10^-4^ | 18% | NT | - |
|  |  | MPN218 | OppF: ABC transporter, peptide/nickel | 851 | N | 1.5×10^-2^ | 11% | NT | - |
|  |  | MPN390 | PdhD: Dihydrolipoamide dehydrogenase | 457 | N | 3.4×10^-8^ | 23% | f^N^ | - |
|  |  | MPN391 | PdhC: Pyruvate dehydrogenase E2 | 402 | N | 6.9×10^-2^ | 13% | f^N^ | - |
|  |  | MPN392 | PdhB: Pyruvate dehydrogenase E1-b | 327 | N | 2.5×10^-9^ | 37% | NT | - |
|  |  | MPN393 | PdhA: Pyruvate dehydrogenase E1-a | 358 | N | 3.8×10^-8^ | 31% | NT | - |
|  |  | MPN394 | Nox: NADH dehydrogenase | 479 | N | 7.0×10^-4^ | 13% | n^N^ | - |
|  |  | MPN430 | Gap: NADP+ | 337 | N | 6.6×10^-5^ | 37% | d^N^ | - |
|  |  | MPN434 | DnaK: Molecular chaperon | 595 | N | 2.5×10^-5^ | 32% | d^N^ | - |
|  |  | MPN470 | PepX: X-Pro dipeptidase | 354 | N | 2.6×10^-2^ | 20% | n^N^ | - |
|  |  | MPN515 | RpoC: DNA-directed RNA polymerase b' | 1290 | N | 2.9×10^-5^ | 10% | n^N^ | - |
|  |  | MPN516 | RpoB: DNA-directed RNA polymerase b | 1391 | N | 3.2×10^-6^ | 9% | n^N^ | - |
|  |  | MPN573 | GroEL: Chaperonin | 543 | N | 2.6×10^-9^ | 48% | n^N^ | - |
|  |  | MPN576 | GlyA: Glycine hydroxymethyltransferase | 406 | N | 2.3×10^-4^ | 17% | NT | - |
|  |  | MPN598 | AtpD: F0F1 ATP synthase b | 475 | N | 6.4×10^-7^ | 33% | d^N^ | - |
|  |  | MPN600 | AtpA: F0F1 ATP synthase a | 518 | N | 1.8×10^-4^ | 17% | d^N^ | - |
|  |  | MPN665 | Tuf: Elongation factor Tu | 394 | N | 2.3×10^-5^ | 43% | NT | - |
|  |  | MPN671 | FtsH: Cell division protein FtsH | 709 | N | 1.9×10^-2^ | 21% | d^N^ | - |

^*^Proteins are listed in five categories as defined in the text.

^†^Molar ratio of proteins in the core fraction was estimated by band densitometry.

^‡^Protein identification of focused band in the core fraction by PMF. Exp.: exception, the maximum value allowed for sequences. Cov.: coverage, the ratio of the portion of the protein sequence covered by matched peptides to the whole sequence length.

^§^Proteins found only in the core-rich fraction, in both the core-rich and soluble fractions, and not in the core-rich fraction are marked "S", "N", and "ND", respectively.

^¶^Localization of the EYFP signal in living cells was categorized into four groups as shown in S2 Fig. NT: Not tested.
