# Supplementary material for: Systematic Structural Analyses of Attachment Organelle in Mycoplasma pneumoniae
Source: PLoS Pathog. 2015 Dec 3;11(12):e1005299. doi: 10.1371/journal.ppat.1005299 (PMC4669176; doi:10.1371/journal.ppat.1005299)
Supplement: S2 Table — (DOCX) [file ppat.1005299.s008.docx]

**Table S2.**  Plasmids used in this study

| ORF clone plasmids (Gateway Entry clones) | | | |  | Tn*4001* plasmids | | | | | |
| --- | --- | --- | --- | --- | --- | --- | --- | --- | --- | --- |
| Plasmid | Marker | Gene | Reference |  | Plasmid | Marker | Promoter | Gene | Localization | Reference |
| pENT-MPN066 | Km^r^ | *CspG* | This work |  | pTK165-D | Ap^r^ Gm^r^ Cm^r^ | *tuf* | *eyfp* | NT | ([1](#_ENREF_1)) |
| pENT-MPN119 | Km^r^ | *topJ* | This work |  | pTK165-MPN066 | Ap^r^ Gm^r^ | *tuf* | *eyfp-cspG* | n | This work |
| pENT-MPN140 | Km^r^ | *orf4* | This work |  | pTK165-MPN119 | Ap^r^ Gm^r^ | *tuf* | *eyfp-topJ* | a, f | This work |
| pENT-MPN141 | Km^r^ | *p1* | This work |  | pTK165-MPN140 | Ap^r^ Gm^r^ | *tuf* | *eyfp-orf4* | d | This work |
| pENT-MPN142 | Km^r^ | *orf6* | This work |  | pTK165-MPN141 | Ap^r^ Gm^r^ | *tuf* | *eyfp-p1* | d | This work |
| pENT-MPN142b | Km^r^ | *p90* | This work |  | pTK165-MPN142 | Ap^r^ Gm^r^ | *tuf* | *eyfp-orf6* | d | This work |
| pENT-MPN295 | Km^r^ | MPN295 | This work |  | pTK165-MPN142b | Ap^r^ Gm^r^ | *tuf* | *eyfp-p90* | d | This work |
| pENT-MPN309 | Km^r^ | *p65* | This work |  | pTK165-MPN295 | Ap^r^ Gm^r^ | *tuf* | *eyfp-*MPN295 | f | This work |
| pMPN310-E | Km^r^ | *hmw2* | ([1](#_ENREF_1)) |  | pTK162 | Ap^r^ Gm^r^ | *tuf* | *eyfp-p65* | a | ([1](#_ENREF_1)) |
| pMPN311-E | Km^r^ | *p41* | ([1](#_ENREF_1)) |  | pMPN310-tuf | Ap^r^ Gm^r^ | *tuf* | *eyfp-hmw2* | a | ([1](#_ENREF_1)) |
| pMPN312-E | Km^r^ | *p24* | ([1](#_ENREF_1)) |  | pMPN311-tuf | Ap^r^ Gm^r^ | *tuf* | *eyfp-p41* | a | ([1](#_ENREF_1)) |
| pENT-MPN332 | Km^r^ | *lon* | This work |  | pMPN312-tuf | Ap^r^ Gm^r^ | *tuf* | *eyfp-p24* | a | ([1](#_ENREF_1)) |
| pENT-MPN372 | Km^r^ | MPN372 | This work |  | pTK165-MPN332 | Ap^r^ Gm^r^ | *tuf* | *eyfp-lon* | a | This work |
| pENT-MPN387 | Km^r^ | MPN387 | This work |  | pTK165-MPN372 | Ap^r^ Gm^r^ | *tuf* | *eyfp-*MPN372 | d | This work |
| pENT-MPN390 | Km^r^ | *pdhD* | This work |  | pTK165-MPN387 | Ap^r^ Gm^r^ | *tuf* | *eyfp-*MPN387 | a | This work |
| pENT-MPN391 | Km^r^ | *pdhC* | This work |  | pTK165-MPN390 | Ap^r^ Gm^r^ | *tuf* | *eyfp-pdhD* | f | This work |
| pENT-MPN394 | Km^r^ | *nox* | This work |  | pTK165-MPN391 | Ap^r^ Gm^r^ | *tuf* | *eyfp-pdhC* | f | This work |
| pENT-MPN430 | Km^r^ | *gap* | This work |  | pTK165-MPN394 | Ap^r^ Gm^r^ | *tuf* | *eyfp-nox* | n | This work |
| pENT-MPN434 | Km^r^ | *dnaK* | This work |  | pTK165-MPN430 | Ap^r^ Gm^r^ | *tuf* | *eyfp-gap* | d | This work |
| pENT-MPN447 | Km^r^ | *hmw1* | This work |  | pTK165-MPN434 | Ap^r^ Gm^r^ | *tuf* | *eyfp-dnaK* | d | This work |
| pENT-MPN452 | Km^r^ | *hmw3* | This work |  | pTK165-MPN447 | Ap^r^ Gm^r^ | *tuf* | *eyfp-hmw1* | a | This work |
| pENT-MPN453 | Km^r^ | *p30* | This work |  | pTK165-MPN452 | Ap^r^ Gm^r^ | *tuf* | *eyfp-hmw3* | a | This work |
| pENT-MPN470 | Km^r^ | *pepX* | This work |  | pTK165-MPN453 | Ap^r^ Gm^r^ | *tuf* | *eyfp-p30* | n | This work |
| pENT-MPN515 | Km^r^ | *rpoC* | This work |  | pTK165-MPN470 | Ap^r^ Gm^r^ | *tuf* | *eyfp-pepX* | n | This work |
| pENT-MPN516 | Km^r^ | *rpoB* | This work |  | pTK165-MPN515 | Ap^r^ Gm^r^ | *tuf* | *eyfp-rpoC* | n | This work |
| pENT-MPN567 | Km^r^ | *p200* | This work |  | pTK165-MPN516 | Ap^r^ Gm^r^ | *tuf* | *eyfp-rpoB* | n | This work |
| pENT-MPN573 | Km^r^ | *groEL* | This work |  | pTK165-MPN567 | Ap^r^ Gm^r^ | *tuf* | *eyfp-p200* | a, f | This work |
| pENT-MPN598 | Km^r^ | *atpD* | This work |  | pTK165-MPN573 | Ap^r^ Gm^r^ | *tuf* | *eyfp-groEL* | n | This work |
| pENT-MPN600 | Km^r^ | *atpA* | This work |  | pTK165-MPN598 | Ap^r^ Gm^r^ | *tuf* | *eyfp-atpD* | d | This work |
| pENT-MPN627 | Km^r^ | *ptsI* | This work |  | pTK165-MPN600 | Ap^r^ Gm^r^ | *tuf* | *eyfp-atpA* | d | This work |
| pENT-MPN671 | Km^r^ | *ftsH* | This work |  | pTK165-MPN627 | Ap^r^ Gm^r^ | *tuf* | *eyfp-ptsI* | d | This work |
|  |  |  |  |  | pTK165-MPN671 | Ap^r^ Gm^r^ | *tuf* | *eyfp-ftsH* | d | This work |
|  |  |  |  |  | pTK170-D | Ap^r^ Gm^r^ Cm^r^ | *tuf* | *eyfp* | NT | This work |
|  |  |  |  |  | pTK170-MPN066 | Ap^r^ Gm^r^ | *tuf* | *cspG-eyfp* | a | This work |
|  |  |  |  |  | pTK170-MPN140 | Ap^r^ Gm^r^ | *tuf* | *orf4-eyfp* | d | This work |
|  |  |  |  |  | pTK170-MPN141 | Ap^r^ Gm^r^ | *tuf* | *p1-eyfp* | a | This work |
|  |  |  |  |  | pTK170-MPN142 | Ap^r^ Gm^r^ | *tuf* | *orf6-eyfp* | n | This work |
|  |  |  |  |  | pTK170-MPN295 | Ap^r^ Gm^r^ | *tuf* | MPN295*-eyfp* | f | This work |
|  |  |  |  |  | pTK170-MPN310 | Ap^r^ Gm^r^ | *tuf* | *hmw2-eyfp* | a | This work |
|  |  |  |  |  | pTK170-MPN372 | Ap^r^ Gm^r^ | *tuf* | MPN372*-eyfp* | d | This work |
|  |  |  |  |  | pTK170-MPN453 | Ap^r^ Gm^r^ | *tuf* | *p30-eyfp* | a | This work |
|  |  |  |  |  | pTK170-MPN627 | Ap^r^ Gm^r^ | *tuf* | *ptsI-eyfp* | n | This work |
|  |  |  |  |  | pTK207-D | Ap^r^ Cm^r^ | *tuf* | *ecfp* | NT | ([1](#_ENREF_1)) |
|  |  |  |  |  | pTK210 | Ap^r^ Cm^r^ | *tuf* | *ecfp-p65* | a | ([1](#_ENREF_1)) |
|  |  |  |  |  | pTK207-MPN310 | Ap^r^ Cm^r^ | *tuf* | *ecfp-hmw2* | a | This work |
|  |  |  |  |  | pTK207-MPN311 | Ap^r^ Cm^r^ | *tuf* | *ecfp-p41* | a | This work |
|  |  |  |  |  | pTK207-MPN312 | Ap^r^ Cm^r^ | *tuf* | *ecfp-p24* | a | This work |
|  |  |  |  |  | pTK207-MPN447 | Ap^r^ Cm^r^ | *tuf* | *ecfp-hmw1* | a | This work |
|  |  |  |  |  | pTK207-MPN452 | Ap^r^ Cm^r^ | *tuf* | *ecfp-hmw3* | a | This work |
|  |  |  |  |  | pTK207-MPN567 | Ap^r^ Cm^r^ | *tuf* | *ecfp-p200* | a,f | This work |

1. Kenri T*, et al.* (2004) Use of fluorescent-protein tagging to determine the subcellular localization of *Mycoplasma pneumoniae* proteins encoded by the cytadherence regulatory locus. *J Bacteriol* 186:6944-6955.
