# Supplementary material for: Systematic Structural Analyses of Attachment Organelle in Mycoplasma pneumoniae
Source: PLoS Pathog. 2015 Dec 3;11(12):e1005299. doi: 10.1371/journal.ppat.1005299 (PMC4669176; doi:10.1371/journal.ppat.1005299)
Supplement: S3 Table — (Upper) Tests for signal positions obtained from single labeling. (Lower) Tests for distances obtained from double labeling. The significant difference between a pair of distances including the same protein may support the positional difference between the other two proteins. The p-values less than 0.05 showing significant difference are colored red. (DOCX) [file ppat.1005299.s009.docx]

|  | P30^C^ | P65^N^ | HMW2^N^ | HMW3^N^ | P1^IF^ | CspG^C^ | HMW1^N^ | MPN387^N^ | HMW2^C^ | P41^N^ | P200^N^ | TopJ^N^ | P24^N^ | Lon^N^ |
| --- | --- | --- | --- | --- | --- | --- | --- | --- | --- | --- | --- | --- | --- | --- |
| P30^C^ |  | 0.0061 | 0.0008 | 10^-4^> | 10^-4^> | 10^-4^> | 10^-4^> | 10^-4^> | 10^-4^> | 10^-4^> | 10^-4^> | 10^-4^> | 10^-4^> | 10^-4^> |
| P65^N^ |  |  | 0.5723 | 0.1179 | 10^-4^> | 10^-4^> | 10^-4^> | 10^-4^> | 10^-4^> | 10^-4^> | 10^-4^> | 10^-4^> | 10^-4^> | 10^-4^> |
| HMW2^N^ |  |  |  | 0.3173 | 10^-4^> | 10^-4^> | 10^-4^> | 10^-4^> | 10^-4^> | 10^-4^> | 10^-4^> | 10^-4^> | 10^-4^> | 10^-4^> |
| HMW3^N^ |  |  |  |  | 0.0018 | 0.0005 | 10^-4^> | 10^-4^> | 10^-4^> | 10^-4^> | 10^-4^> | 10^-4^> | 10^-4^> | 10^-4^> |
| P1^IF^ |  |  |  |  |  | 0.5957 | 0.2119 | 10^-4^> | 10^-4^> | 10^-4^> | 10^-4^> | 10^-4^> | 10^-4^> | 10^-4^> |
| CspG^C^ |  |  |  |  |  |  | 0.4761 | 10^-4^> | 10^-4^> | 10^-4^> | 10^-4^> | 10^-4^> | 10^-4^> | 10^-4^> |
| HMW1^N^ |  |  |  |  |  |  |  | 10^-4^> | 10^-4^> | 10^-4^> | 10^-4^> | 10^-4^> | 10^-4^> | 10^-4^> |
| MPN387^N^ |  |  |  |  |  |  |  |  | 0.9845 | 0.5988 | 0.5077 | 0.0116 | 0.0011 | 10^-4^> |
| HMW2^C^ |  |  |  |  |  |  |  |  |  | 0.6698 | 0.5742 | 0.0267 | 0.0049 | 10^-4^> |
| P41^N^ |  |  |  |  |  |  |  |  |  |  | 0.8524 | 0.0472 | 0.0084 | 10^-4^> |
| P200^N^ |  |  |  |  |  |  |  |  |  |  |  | 0.0953 | 0.0255 | 10^-4^> |
| TopJ^N^ |  |  |  |  |  |  |  |  |  |  |  |  | 0.6375 | 0.0016 |
| P24^N^ |  |  |  |  |  |  |  |  |  |  |  |  |  | 0.0029 |
| Lon^N^ |  |  |  |  |  |  |  |  |  |  |  |  |  |  |

|  | P30^C^ - P65^N^ | P30^C^ - HMW2^N^ | P30^C^ - HMW3^N^ | P65^N^ - HMW2^N^ | P65^N^ - HMW3^N^ | HMW2^N^ - HMW3^N^ |
| --- | --- | --- | --- | --- | --- | --- |
| P30^C^ - P65^N^ |  | 0.0006 | 10^-4^> | 10^-4^> | 10^-4^> |  |
| P30^C^ - HMW2^N^ |  |  | 0.0330 | 0.2657 |  | 10^-4^> |
| P30^C^ - HMW3^N^ |  |  |  |  | 0.7536 | 0.0044 |
| P65^N^ - HMW2^N^ |  |  |  |  | 0.0953 | 10^-4^> |
| P65^N^ - HMW3^N^ |  |  |  |  |  | 0.0012 |
| HMW2^N^ - HMW3^N^ |  |  |  |  |  |  |
